# Integrative Multi-Omics Reveals Tissue-Specific Postharvest Changes in Blueberry Fruit

**DOI:** 10.1021/acs.jafc.6c13073

**Published:** 2026-10-06

**Authors:** Molla Fentie Mengist, Romit Seth, Massimo Iorizzo

**Affiliations:** † Agricultural Research Station, Virginia State University, Petersburg, Virginia 23806, United States; ‡ Plants for Human Health Institute, North Carolina State University, Kannapolis, North Carolina 28081, United States; § Department of Horticultural Science, North Carolina State University, Raleigh, North Carolina 27607, United States

**Keywords:** flavonoids, sugars and amino acids, cold stress, transcriptome, metabolome

## Abstract

Postharvest cold storage is essential for maintaining blueberry fruit quality, yet the molecular basis of tissue-specific responses during storage remains poorly understood. Here, we integrated widely targeted metabolomics and transcriptomics to characterize peel and pulp responses during 6 weeks of storage at 4 °C. Metabolomic profiles revealed pronounced tissue specialization, with peel enriched in flavonoids, terpenoids, and phenylpropanoid-derived metabolites, whereas pulp accumulated amino acids and other primary metabolites. In contrast, transcriptomic variation was dominated by storage, indicating a largely conserved response across tissues. Multi-omics analyses using DIABLO, MOFA2, and network analysis identified 65 genes and 58 metabolites associated with storage-related and tissue-specific variation. These analyses highlighted jasmonate- and oxylipin-associated pathways, antioxidant responses, membrane lipid remodeling, and phenylpropanoid metabolism, alongside reduced expression of aquaporins and cell wall-modifying genes. Overall, these findings provide a systems-level view of blueberry storage responses and identify candidate molecular processes associated with postharvest quality and storage performance.

## Graphical abstract

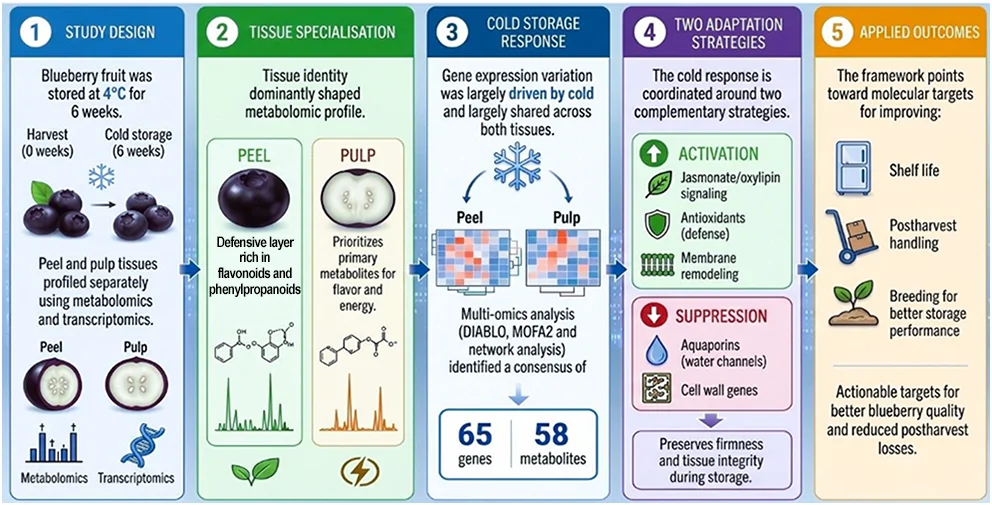

## 1 Introduction

Blueberries (*Vaccinium spp*) are recognized as rich sources of health-promoting bioactive metabolites.^1–5^ They contain a diverse array of bioactive compounds, including anthocyanins, flavonoids, phenolic acids, and organic acids, which enhance fruit quality attributes, such as color, flavor, and overall sensory appeal.^6,7^ Beyond sensory appeal, clinical and epidemiological evidence support the potential health benefits of these compounds, including improving cognitive function, reducing cardiovascular risk, and managing type 2 diabetes.^2–4^ Given rising consumer demand and increasing market value, maintaining fruit postharvest quality poses a significant challenge for both the blueberry industry and modern breeding programs.^8–13^ The distribution of metabolites varies among different tissues in various fruits such as blueberries. Studies have shown that the peel and pulp have unique metabolic profiles and serve different functions. The peels are high in anthocyanins and phenolic compounds, whereas the pulp contains sugars and organic acids, contributing to its sweetness and acidity.^14,15^ While these studies establish the importance of tissue-specific contributions to fruit quality, they are largely limited to measurements at harvest and typically focus on a few sets of metabolites. As a result, they provide a static view of the tissue specialization. It remains unclear how peel and pulp metabolomes change dynamically during postharvest storage, how these networks of primary and secondary metabolites are coordinated across tissues, and how these metabolomic changes are regulated at the transcriptional level.

Cold storage is the main method used to extend the shelf life of blueberries by slowing respiration and delaying senescence. While low temperatures help maintain firmness and limit decay, prolonged cold storage can trigger biochemical and physiological changes that can affect the nutritional value and flavor. Previous studies have observed notable shifts in anthocyanins, aromatic compounds, and organic acids in blueberries.^16–19^ However, these studies have generally examined changes at the whole-fruit level, potentially masking tissue-specific responses in peel versus pulp, and have often focused on a limited set of metabolites. As a result, the tissue-specific dynamics of storage-induced metabolic changes remain poorly understood.

In other fruit crops, tissue-specific multi-omics studies have revealed coordinated metabolic and transcriptional networks that regulate ripening, stress responses, and quality traits.^20–23^ These studies reveal that integrating metabolomic and transcriptomic data is essential for linking metabolite changes to the gene regulation. Despite these advances, comparable integrative studies have not yet been applied to blueberries, leaving a knowledge gap in understanding the regulatory mechanisms underlying tissue-specific metabolic changes during postharvest storage. In particular, how metabolic and transcriptional changes are coordinated in peel and pulp during storage and how these processes contribute to postharvest quality decline remain unclear. Addressing this gap requires a high-resolution spatiotemporal framework that integrates metabolomic and transcriptomic layers to capture tissue-specific changes during the storage.

Texture is another important determinant of postharvest fruit quality, and blueberries undergo substantial changes in textural properties during cold storage.^13,24,25^ Changes in textural properties indicate structural and physiological changes in the fruit tissues and therefore provide a phenotypic dimension of postharvest responses. However, the molecular changes associated with these textural responses remain poorly characterized at the tissue-specific level.

In this study, we performed a comprehensive analysis of the metabolomic and transcriptomic changes in the blueberry peel and pulp at harvest and during cold storage. We additionally evaluated fruit texture to examine patterns of texture changes, alongside metabolite and gene expression responses. Specifically, we characterize tissue-specific metabolomic changes during storage, identify transcriptomic shifts associated with storage-induced metabolic responses, and integrate metabolomic and transcriptomic data sets to uncover pathways underpinning postharvest quality changes. We further examined whether these molecular responses were associated with changes in textural-related parameters during storage.

To our knowledge, this is the first study in blueberries to combine tissue-specific resolution (peel vs pulp), temporal analysis across postharvest storage, and multi-omics integration, providing new insights into the molecular mechanisms driving fruit quality and highlighting potential targets for postharvest management and future improvement efforts.

## 2 Materials and Methods

### 2.1 Materials and Sample Preparation

Blueberries (*Vaccinium corymbosum* L., cv. “Duke”) were harvested at full maturity from the Virginia State University Randolph Farm (Petersburg, VA, USA). The “Duke” cultivar was selected due to its commercial dominance and well-documented postharvest susceptibility to textural softening and metabolic alterations.^12^ Three independent biological replicates were collected from distinct plants. Each biological replicate comprised a pool of ∼5 to 10 uniform, disease-free berries harvested from individual bushes.

For postharvest storage, berries were packed into commercial perforated polyethylene terephthalate (PET) clamshell containers and placed in dark cold storage maintained at 4 °C and 95% relative humidity (RH) under ambient atmospheric conditions (no atmospheric modification). Samples were harvested and evaluated at baseline (T0, harvest) and after 6 weeks of storage (T1, 4 °C), representing two distinct physiological stages of postharvest cold adaptation.^12,13^ At each point, peel and pulp were carefully separated using a sterile blade and immediately flash-frozen in liquid nitrogen. Tissues designated for metabolomic analysis were stored at −80 °C, then lyophilized, finely ground, and kept at −20 °C until metabolite extraction. Tissues designated for RNA-seq analysis were stored directly at −80 °C until RNA extraction.^12^

### 2.2 Texture Profiling

For texture analysis, fully ripened blueberries free of visible defects or disease were placed in 188 mL plastic portion cups (Uline, Pleasant Prairie, WI, USA) with lids containing five evenly spaced 3 mm ventilation holes. Cups were arranged on shallow cardboard trays, enclosed in large plastic bags, and stored at 4 °C and 95% relative humidity. Fruits showing visible mold during storage were excluded from analysis. Texture was measured using a TA.XTPlus texture analyzer (Stable Micro Systems, Hamilton, MA, USA) following the protocol described by.^12,13,24,26^ A 2 mm flat cylindrical probe (TA-52) performed a penetration test along the fruit equator at a speed of 1 mm s^–1^ to 80% strain, with a trigger force of 0.5 N and a data acquisition rate of 100 points s^–1^. The procedure generated 18 mechanical texture parameters (Table S1).

### 2.3 Widely Targeted Metabolomic Profiling

Metabolite profiling was conducted using the Plant Widely Targeted Metabolomics Service provided by Metware Biotechnology (Woburn, MA, USA). Metabolite extraction from lyophilized samples was performed as described.^12^ Briefly, 50 mg of freeze-dried tissue powder was homogenized in 1.2 mL of prechilled (−20 °C) 70% aqueous methanol containing internal standards. The mixture was vortexed six times for 30 s at 30 min intervals to ensure efficient metabolite extraction and centrifuged at 12,000 rpm for 3 min at 4 °C. The resulting supernatants were collected and filtered through a 0.22 μm hydrophilic PTFE membrane before UPLC-MS/MS analysis.

Chromatographic separation was carried out using an ExionLC AD UPLC system equipped with an Agilent SB-C18 column (1.8 μm, 2.1 mm × 100 mm) maintained at 40 °C. The mobile phases consisted of (A) ultrapure water containing 0.1% formic acid and (B) LC-MS grade acetonitrile containing 0.1% formic acid. The gradient elution program was set as follows: 5% B at 0.00 min, linear increase to 95% B over 9.00 min, held at 95% B for 1.00 min (9.00–10.00 min), returned to 5% B from 10.00 to 11.10 min, and re-equilibrated at 5% B until 14.00 min. The flow rate was 0.35 mL/min with an injection volume of 2 μL.

Metabolite profiling was performed using a TripleTOF 6600+ quadrupole time-of-flight mass spectrometer (SCIEX). The source temperature was 500 °C, with ion spray voltages of +5500 V and −4500 V in positive and negative ion modes, respectively. Ion source gases 1 and 2 were set at 50 and 60 psi, respectively, and curtain gas was set at 25 psi. The declustering potential (DP) was 80 V and −80 V, and the collision energy (CE) was 30 V and −30 V in positive and negative ion modes, respectively, with a collision energy spread of 15 V. Metabolites were identified using Metware’s in-house metabolite database (MWDB) based on accurate precursor mass, MS/MS fragmentation patterns, isotopic distribution, and retention time (RT). Redundant ion signals, including isotopic peaks, common adducts (K^+^, Na^+^, and NH_4_^+^), and fragment signals derived from higher-molecular-weight precursors, were removed during data processing. According to Metware’s identification criteria, metabolites were assigned to three substance identification levels: Level 1, for compounds with matching scores for MS/MS spectrometry, RT, and database material greater than 0.7; Level 2, for compounds with matching scores between 0.5 and 0.7; and Level 3, for compounds for which Q1, Q3, RT, DP, and CE were consistent with the database substance. These categories represent Metware’s substance identification levels and are not presented as formal MSI identification levels.

Relative metabolite abundance was quantified using a QTRAP 6500+ triple quadrupole linear ion-trap mass spectrometer (SCIEX) equipped with an ESI Turbo ion-spray interface and operated in positive and negative ion modes. The source temperature was 550 °C, with ion spray voltages of +5500 V and −4500 V in positive and negative ion modes, respectively. Ion source gases 1 and 2 were set at 50 and 60 psi, respectively, and curtain gas was set at 25 psi. Metabolites were quantified using multiple reaction monitoring (MRM), with compound-specific DP and CE values optimized for each MRM ion pair. Mass spectrometry data were processed using MultiQuant software. Chromatographic peaks were integrated and corrected, and peak area was used as a measure of the relative abundance of each metabolite across samples. Missing values were imputed using one-fifth of the minimum value for each metabolite across samples. Quality control (QC) samples were prepared by mixing sample extracts and were inserted approximately every 10 test samples to assess analytical reproducibility. Metabolites with a QC coefficient of variation (CV) > 0.50 were excluded from downstream statistical analyses.

### 2.4 Data Preprocessing and Statistical Analyses

Metabolomic data from peel and pulp tissues at T0 and T1 were processed in R. Raw data were log_2_-transformed to stabilize variance, and features with no variation across treatments were removed. Nonmetric multidimensional scaling (NMDS) ordination was performed using Bray–Curtis dissimilarity with two dimensions (*k* = 2) and 100 permutations (trymax = 100) on unique sample profiles to evaluate differences in metabolite composition among groups. Global metabolic variation was further assessed using principal component analysis (PCA), which summarized overall compositional differences and highlighted features contributing to the first two principal components.

To test the effects of tissue type, storage duration, and their interaction on metabolic profiles, a permutational multivariate analysis of variance (PERMANOVA) was performed. A linear modeling framework implemented in the LIMMA package, incorporating empirical Bayes moderation, was used to identify differentially accumulated metabolites (DAMs) across tissues and storage durations. Metabolites with an absolute log_2_ fold change ≥2 and an adjusted *p*-value <0.05 were considered statistically significant. Significant features identified from individual contrasts were integrated to assess overlap using Venn diagrams. Multivariate and exploratory analyses, including NMDS, volcano plots, PCA, and correlation heatmaps, were performed using ggplot2 and ComplexHeatmap. Pathway enrichment analysis was conducted using MetaboAnalystR, applying over-representation analysis based on the hypergeometric test, coupled with pathway topology analysis using relative betweenness centrality.

### 2.5 Transcriptome Profiling and Functional Analysis

Total RNA was isolated from partitioned peel and pulp tissues using the Spectrum Plant Total RNA Kit (Sigma-Aldrich, St. Louis, MO, USA) according to the manufacturer’s instructions. RNA integrity and concentration were verified before downstream processing. Library construction and high-throughput sequencing were performed by Novogene (Sacramento, CA, USA). For library generation, RNA underwent either poly­(A) selection or rRNA depletion, followed by the synthesis of cDNA. Strand-specific RNA sequencing was performed using a 150 bp paired-end approach on the Illumina NovaSeq 6000 platform.

The raw sequence data underwent rigorous preprocessing using Trimmomatic v0.39 ^27^ to eliminate adapter sequences and filter out low-quality reads (default parameters). The cleaned, high-quality reads were aligned to the W85_v2 blueberry reference genome^28^ using the splice-aware aligner STAR v2.7.10a.^29^ Transcript abundances were then quantified using StringTie v1.3.6 ^30^

Differential expression genes (DEGs) analysis was performed using the DESeq 2 package.^31^ Raw count data were first filtered to retain genes with a minimum expression threshold of at least 10 counts in at least two samples to remove lowly expressed features. Following model fitting, statistical significance was assessed using Wald tests with *p*-values adjusted for multiple testing using the Benjamini–Hochberg false discovery rate (FDR) procedure. Genes were considered differentially expressed if they met stringent high-confidence criteria, including an adjusted p-value (FDR) < 0.01 and an absolute log2 fold change ≥2. To further ensure robustness, genes with low average expression (baseMean <50) were excluded from downstream interpretation. Log2 fold changes were shrunk using the apeglm method to reduce noise and improve effect size estimation, particularly for low-count genes. These combined thresholds were consistently applied across all contrasts to define high-confidence DEGs.

Functional annotation of DEGs was conducted using the manually curated UniProtKB/Swiss-Prot database, with a blueberry-specific reference derived from the Vaccinium Genome Database (*V. caesariense* W85–20 genome assembly). Gene Ontology (GO) terms and corresponding protein identifiers were retrieved from UniProtKB and used directly as input for downstream analyses in the R environment. GO term relationships and mappings were managed using the GO.db package, and redundant or noninformative terms were filtered prior to enrichment analysis. GO enrichment was assessed using a hypergeometric test, with multiple testing correction performed via the Benjamini–Hochberg false discovery rate (FDR) method. GO terms with an adjusted *p*-value (FDR) < 0.05 were considered significantly enriched.

### 2.6 Multi-Omics Integration Analysis

Transcriptomic and metabolomic data sets were integrated using matched sample identifiers. Previously identified DEGs and DAMs were used as inputs for multi-omics integration analyses. Unsupervised integration was performed using MOFA2 (Multi-Omics Factor Analysis), which identifies latent factors that capture shared and modality-specific sources of variation across multiple omics data sets.^32^ Transcriptional and metabolomic matrices were supplied as separate views, and view scaling was enabled to balance contributions from each omics layer. Models were trained using spike-and-slab priors and automatic relevance determination with two latent factors and a maximum of 2000 training iterations. Model convergence, latent factor stability across computational restarts, and the proportion of variance explained (*R* ^2^) per omic layer were recorded. Variance explained, factor scores, and feature weights were extracted, and top-weighted gene and metabolite features were retained as candidate drivers of shared cross-omics variation.^32^

Supervised integration was performed using DIABLO (Data Integration Analysis for Biomarker discovery using Latent Components) implemented in the mixOmics package.^33^ Samples were classified according to combined tissue–time groups, and a two-component sparse block PLS-DA model was fitted using a design matrix linking transcriptomic and metabolomic data sets (design strength = 0.8). To prevent model overfitting and evaluate feature stability, parameter tuning and variable selection were optimized using 10-fold cross-validation repeated 10 times via the tune.block.splsda function, selecting the optimal number of variables per component to minimize classification error rates. Feature selection was optimized by leave-one-out cross-validation when feasible; otherwise, conservative default settings were applied. Features with nonzero loadings were retained as discriminative candidates.

Gene–metabolite associations were assessed using Spearman’s rank correlation across matched samples. *P-*values were adjusted using the Benjamini–Hochberg false discovery rate procedure.^34^ Significant associations (|ρ| ≥ 0.85, adjusted *P* ≤ 0.01) were used to construct a bipartite network. Node degree was used as a measure of network connectivity, and highly connected hub genes and metabolites were identified as features with node degrees within the upper quartile of the degree. Network analyses were performed using the igraph package.^35^

Candidate biological drivers were prioritized by integrating evidence from the MOFA2 feature weights, DIABLO loadings, and network connectivity. Features supported by at least two approaches were designated as consensus candidates. Consensus features were subsequently clustered, and both genes and metabolites were functionally annotated.

To identify coordinated transcriptomic and metabolomic modules, consensus genes and metabolites were clustered separately using hierarchical clustering based on Pearson correlation distances and Ward’s linkage method. Representative module eigengenes, defined as the first principal component of each cluster, were calculated to summarize cluster-level expression and metabolite accumulation patterns. Associations between gene and metabolite modules were evaluated using Pearson correlation analysis of module eigengenes. Significant module relationships were visualized as correlation heatmaps and used to identify coordinated transcriptomic and metabolomic responses associated with tissue type and storage duration.

## 3 Results

### 3.1 Texture

A total of 18 texture parameters were evaluated to characterize changes in fruit textural properties during cold storage (Figure S1). Parameters primarily associated with the fruit skin, including F1MM, FM, DFM, SFM, AFM, and LDFM, showed distinct changes over the storage period, with F1MM, FM, SFM, AFM, and LDFM decreasing, whereas DFM and BuStr increased. Pulp-specific parameters, including AIF, MIF, and NIP, also decreased during storage. The remaining parameters represented combined mechanical responses of the skin and pulp. Overall, the magnitude of change varied among parameters, with fold changes ranging from approximately 1.0- to 6.9-fold between the initial and final storage time points. The largest decreases were observed for Young’s modulus (6.92-fold), YM20 (5.52-fold), and NIP (4.09-fold), while DFM and BuStr increased by 1.17- and 1.06-fold, respectively.

### 3.2 Overview of Metabolome Data Sets

Using a widely targeted metabolomics platform, we identified a total of 2160 metabolites across all samples. These metabolites cover a wide range of chemical classes, including alkaloids, amino acids, flavonoids, lignans, lipids, phenolic acids, organic acids, and others. Among these metabolomic classes, flavonoids were the most abundant, highlighting their prominence in the blueberry metabolome (Figure S2a).

### 3.3 Effect of Tissue and Storage Time on Metabolomic Profiles

Metabolomic profiling revealed that both tissue (peel vs pulp) and postharvest storage significantly influenced metabolite abundance, without significant interaction between the two factors (Table S2), indicating that their effects on metabolite abundance are largely independent.

NMDS ordination demonstrated a clear separation of samples by tissue type and storage time (Figure S2b), with a more pronounced separation between storage treatments in peel compared to pulp, suggesting a stronger cold storage effect in peel tissues. Similarly, PCA revealed distinct clustering of the four experimental groups (peel/pulp × T0/T1), with PC1 and PC2 explaining 49.6% and 23.2% of the total variance, respectively (Figure S2c; Figure S3). Together, these results highlight that both tissue type and postharvest storage duration are key drivers of metabolic variation in blueberry fruit.

#### 3.3.1 Tissue-Specific Metabolic Accumulation

An initial assessment based on raw data percentages without statistical testing indicated pronounced tissue-specific distribution patterns. Flavonoids and terpenoids were predominantly enriched in the peel, whereas alkaloids, amino acids and their derivatives, and lignans were more abundant in the pulp (Figure 1, Table S3). Subsequent statistical analysis confirmed these patterns, revealing that a substantial portion of the metabolome was significantly different between peel and pulp at both T0 (826 metabolites) and T1 (837 metabolites) (Figure 2a,b). These differences were highly consistent across time points, with many metabolites remaining significant under both conditions, as illustrated by the Venn diagram (Figure S4). Notably, tissue-specific variation far exceeded the effects of storage time (Figure 2).

**Figure 1:**
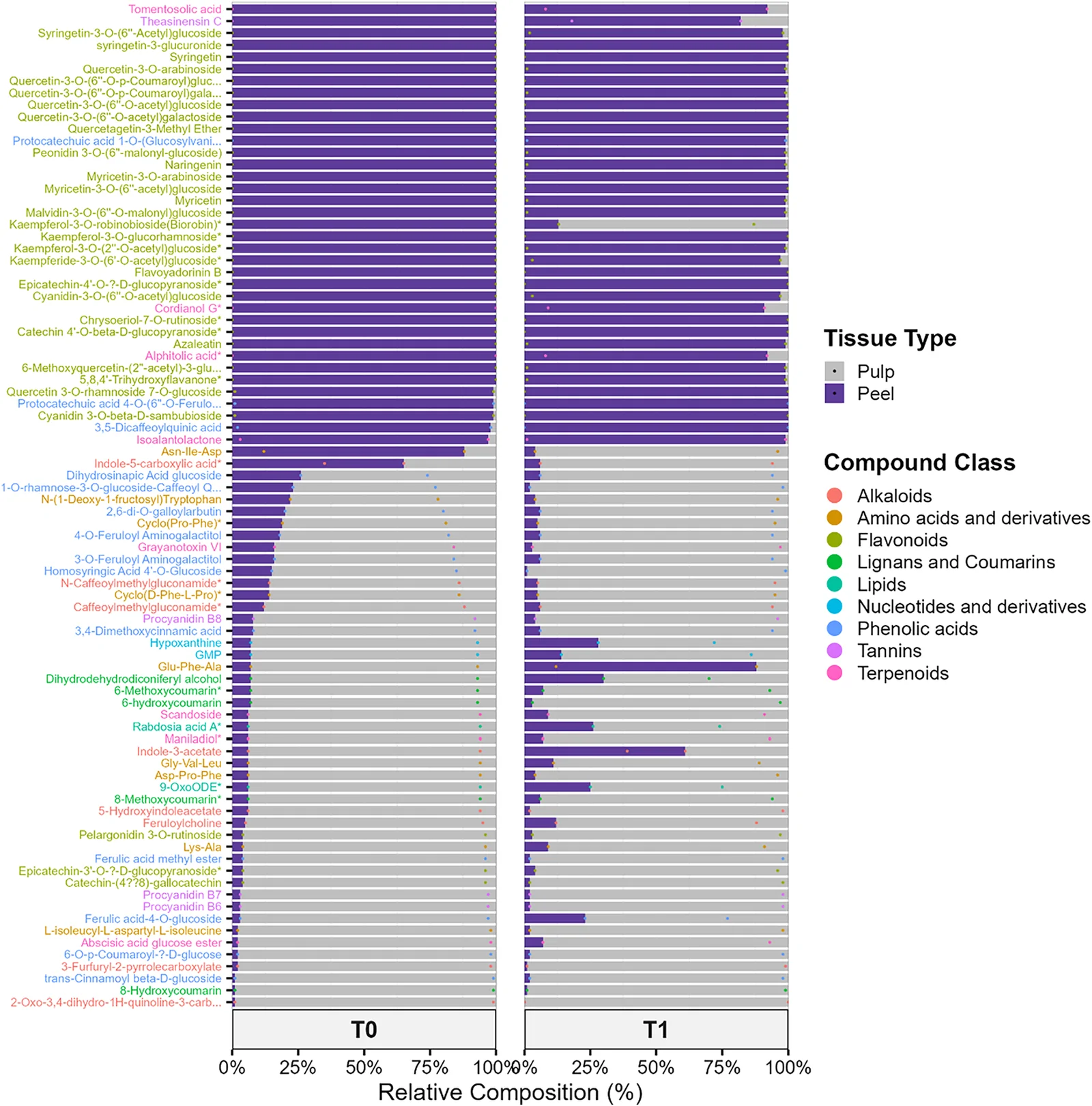
Percentage distribution of metabolite classes in blueberry peel and pulp tissues at harvest (T0) and after 6 weeks of cold storage at 4 °C (T1).

**Figure 2:**
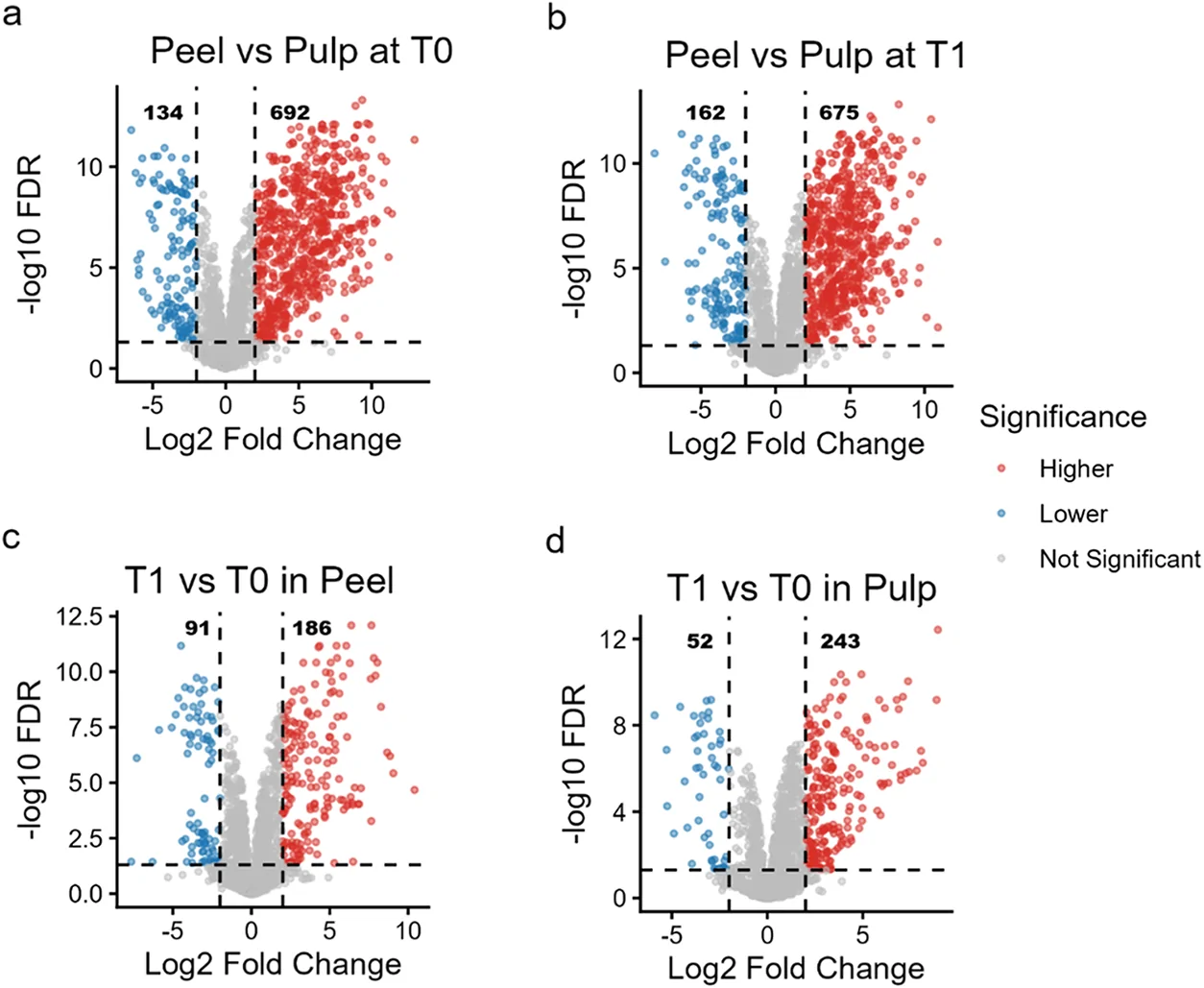
Volcano plots show differential metabolites identified by LIMMA across tissues and storage time points. (a) Comparison between Peel and Pulp at harvest (T0), (b) Peel vs Pulp after 6 weeks of storage (T1), (c) temporal comparison in Peel between T1 and T0, and (d) temporal comparison in Pulp between T1 and T0. Each point represents a metabolite, with the *x*-axis showing log2 fold change and the *y*-axis showing −log10 FDR. Metabolites with log2 fold change ≥2 and FDR < 0.05 are considered significantly up- (orange) or downregulated (cyan), whereas nonsignificant metabolites are shown in gray. The number of up- and downregulated metabolites in each contrast is indicated in the panel title.

Most DAMs were more abundant in the peel than in the pulp (Figure 3), highlighting the peel as a metabolically active outer layer enriched in specialized, likely defense-related compounds. At harvest (T0), this divergence was particularly pronounced: of the 2160 metabolites profiled, 692 (∼32%) were significantly higher in the peel, whereas 134 (∼6%) were higher in the pulp (Figure 2a); nearly 38% of metabolites exhibited differential accumulation. Pathway enrichment analysis revealed that flavonoids, including anthocyanins, flavonols, and related phenylpropanoid intermediates, were strongly enriched in peel tissues (Figure 4a,b). In contrast, the pulp was enriched in sugars and amino acid-related metabolites, particularly glucose, fructose, and derivatives of alanine, serine, and aspartate.

**Figure 3:**
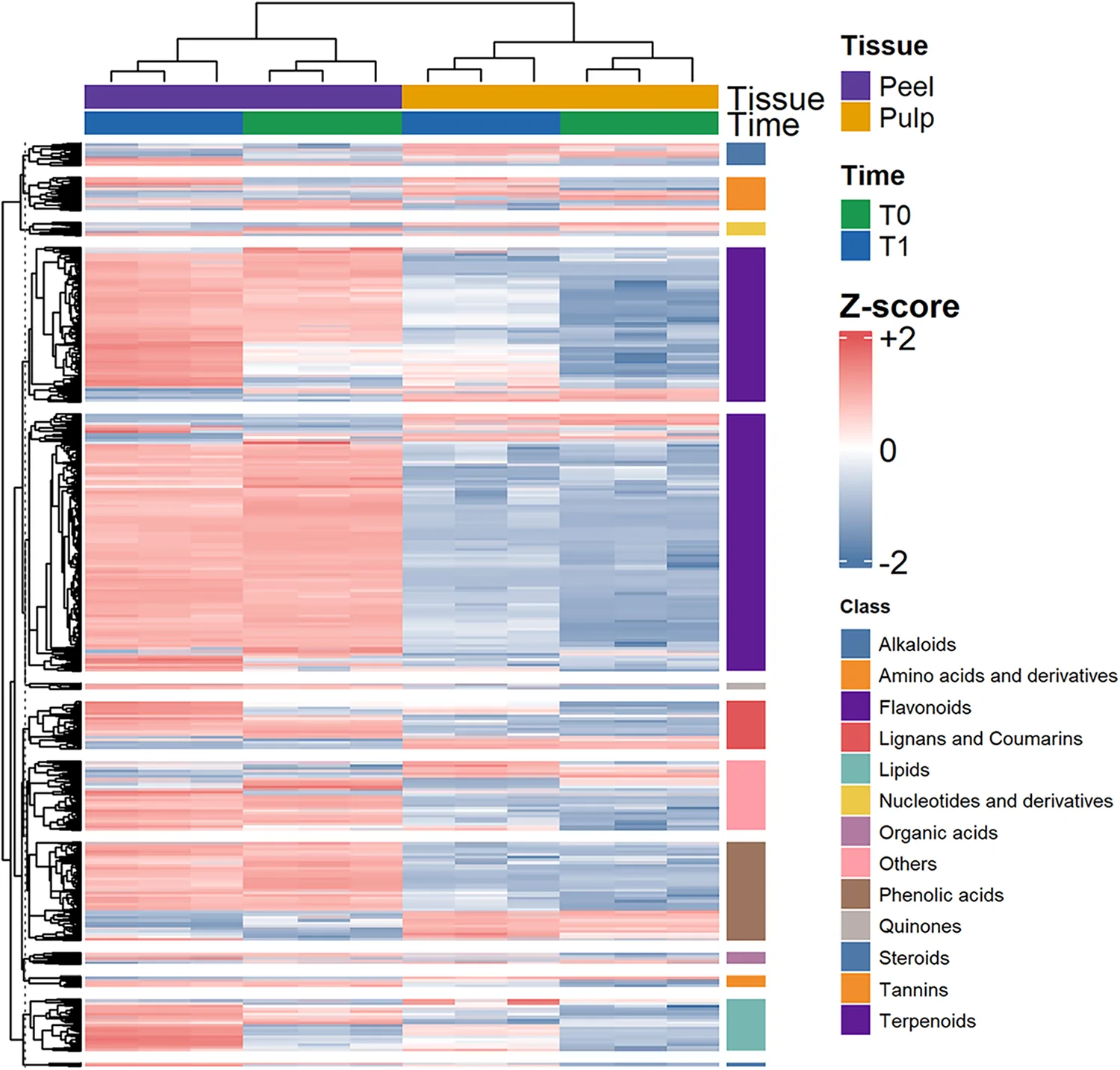
Heatmap showing the scaled abundance patterns of significant metabolites across tissues and storage time points in blueberry. Metabolites were identified as significantly higher or lower by LIMMA (log2 fold change ≥ 2, FDR < 0.05) and are grouped by metabolite Class. Rows represent individual metabolites (clustered within each Class), and columns represent biological replicates, ordered by tissue (Peel, Pulp) and time point (T0 = harvest, T1 = 6 weeks storage). Metabolite abundances were normalized by Z-score per metabolite to highlight relative changes across samples. Column annotations indicate tissue type (Peel: orange, Pulp: cyan) and storage time (T0: teal, T1: coral), while row annotations indicate metabolite Class with distinct colors.

**Figure 4:**
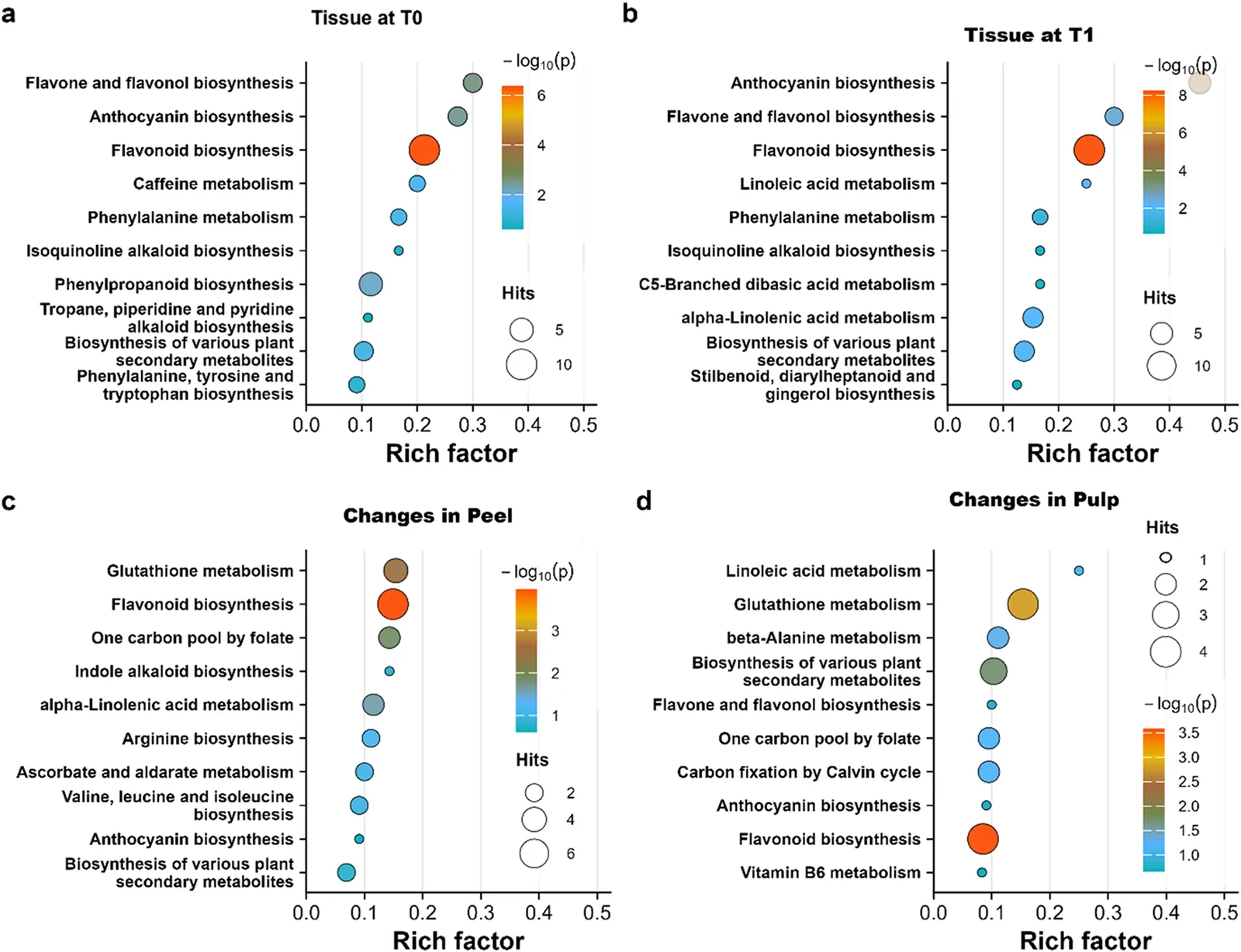
KEGG pathway enrichment analysis of significant metabolites across tissues and storage time points in blueberry. (a) Peel vs Pulp at harvest (T0), (b) Peel vs Pulp after 6 weeks of storage (T1), (c) temporal comparison in Peel between T1 and T0, and (d) temporal comparison in Pulp between T1 and T0. Each point represents a KEGG pathway, with the *x*-axis showing the rich factor (proportion of significant metabolites in the pathway) and the *y*-axis showing pathway names. Point size reflects the number of significant hits in each pathway, and color indicates −log10­(p) from enrichment analysis (higher values indicate greater significance). Pathways are ordered by the rich factor within each panel. This visualization highlights the tissue- and time-specific metabolic pathways most impacted by storage and tissue type, allowing comparison of shared and unique enriched pathways across contrasts.

#### 3.3.2 Storage-Induced Metabolomic Modeling

Cold storage significantly affected the metabolome of both tissues, with 277 metabolites in the peel and 295 in the pulp showing significant changes after 6 weeks (Figure 2c,d). In both tissues, metabolite accumulation predominated over depletion. In the peel, 186 metabolites (∼67%) increased while 91 (∼33%) decreased, whereas in the pulp this trend was more pronounced, with 243 (∼82%) increasing and 52 (∼18%) decreasing (Figure 2c,d).

Pathway enrichment analysis revealed strong induction of amino acid biosynthesis pathways at T1, including valine, leucine, and isoleucine biosynthesis and arginine biosynthesis in the peel and β-alanine metabolism in the pulp (Figure 4c,d). In parallel, glutathione metabolism was enriched in both tissues following storage (Figure 4c,d).

### 3.4 Tissue-Specific and Storage-Dependent Gene Expression

To complement the metabolomic analysis, we performed transcriptomic profiling of blueberry peels and pulp at T0 and T1, capturing genome-wide tissue-specific gene expression dynamics during storage.

#### 3.4.1 Global DEGs Pattern and Transcriptome Dynamics

PCA revealed a clear separation of samples by tissue type and storage duration, indicating distinct transcriptional profiles at harvest and pronounced expression shifts during storage (Figure S5). A total of 6,794 DEGs were identified across all comparisons (Figure 5), reflecting substantial postharvest transcriptomic remodeling. Notably, storage-induced changes (∼2226 DEGs per tissue) were nearly twice the magnitude of baseline tissue differences (∼1235 DEGs at harvest) (Figure 5a–d). The Venn diagram further showed that 54% of cold-induced genes were shared between peel and pulp, whereas only 24% of DEGs were commonly regulated at the tissue-specific level (Figure S6).

**Figure 5:**
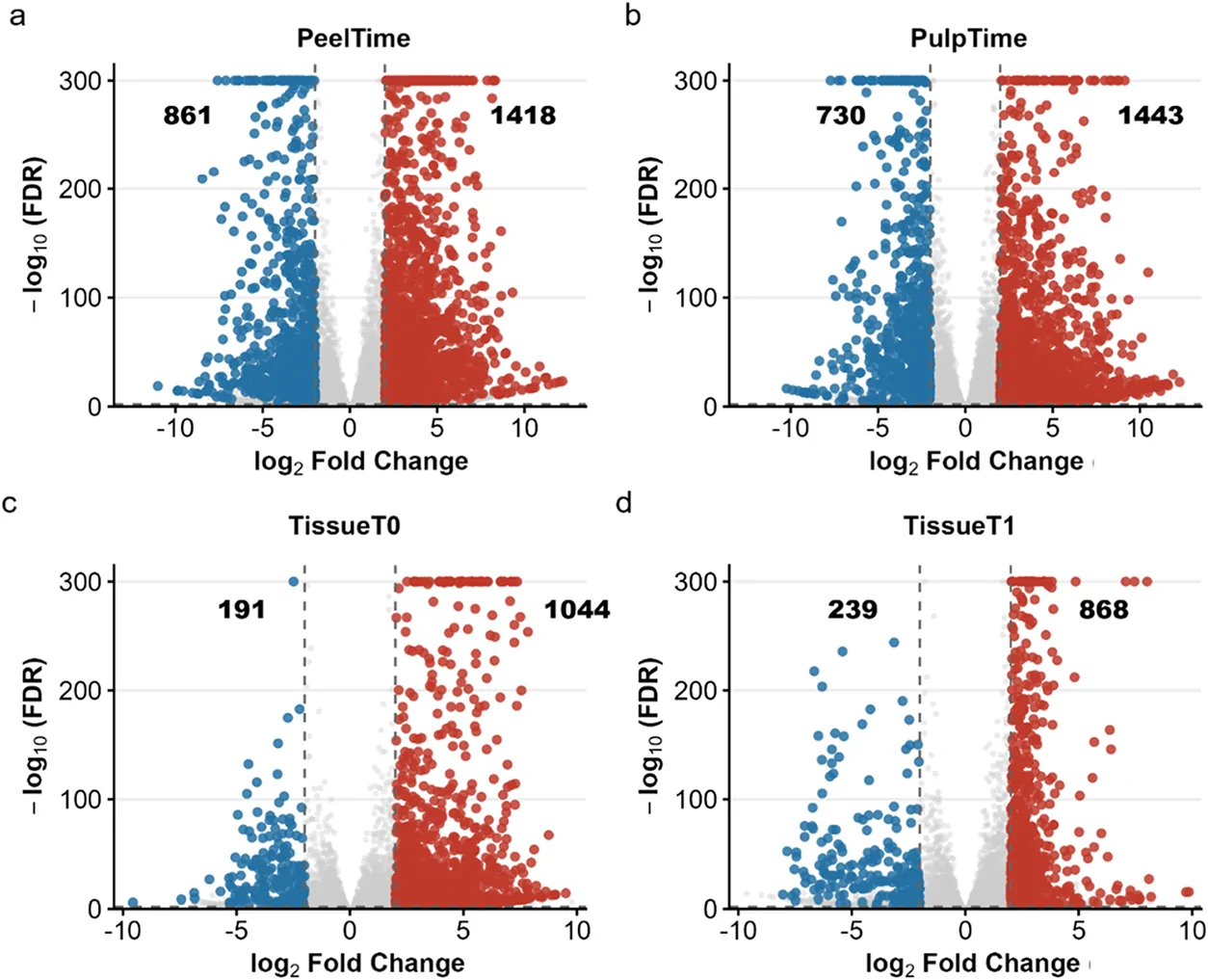
Volcano plots of high-confidence DEGs for selected tissue and storage contrasts. Each point represents a gene, plotted by log2 fold-change (apeglm shrinkage) versus −log10 adjusted *p*-value. Upregulated genes are shown in red, downregulated genes in blue, and nonsignificant genes in gray. Dashed lines indicate fold change and significance thresholds. Top DEGs for each contrast are labeled. Panels (a–d) correspond to different contrasts: (a) Peel_T0 vs Peel_T1, (b) Pulp_T0 vs Pulp_T1, (c) Peel_T0 vs Pulp_T0, and (d) Peel_T1 vs Pulp_T1.

#### 3.4.2 Tissue Specialization and Cold-Induced Transcriptome Changes

Gene Ontology (GO) enrichment analysis revealed a highly consistent transcriptional response in both peel and pulp during cold storage, characterized by activation of a shared antioxidant and defense-related program (Figure 6 a,b). This response was primarily defined by the enrichment of glutathione metabolism, along with processes related to oxidative stress, wounding, and pathogen defense. At the cellular level, apoplast and plasma membrane components were enriched, while molecular functions were dominated by oxidoreductases, UDP-glycosyltransferases, monooxygenases, and chitin-binding activities. The pulp additionally exhibited enrichment of cell wall organization and fundamental defense pathways. At harvest (T0), the peel showed strong enrichment of cutin, wax, and flavonoid biosynthetic pathways, photosystem-related components, and fatty acid elongation activities relative to the pulp (Figure 6c). Following prolonged cold storage (T1), tissue-specific differences expanded (Figure 6d) to include lipid-associated processes (lipid droplet and lipid storage body components), flavonoid biosynthesis, reserve mobilization pathways, nutrient reservoir activity, and ferritin binding.

**Figure 6:**
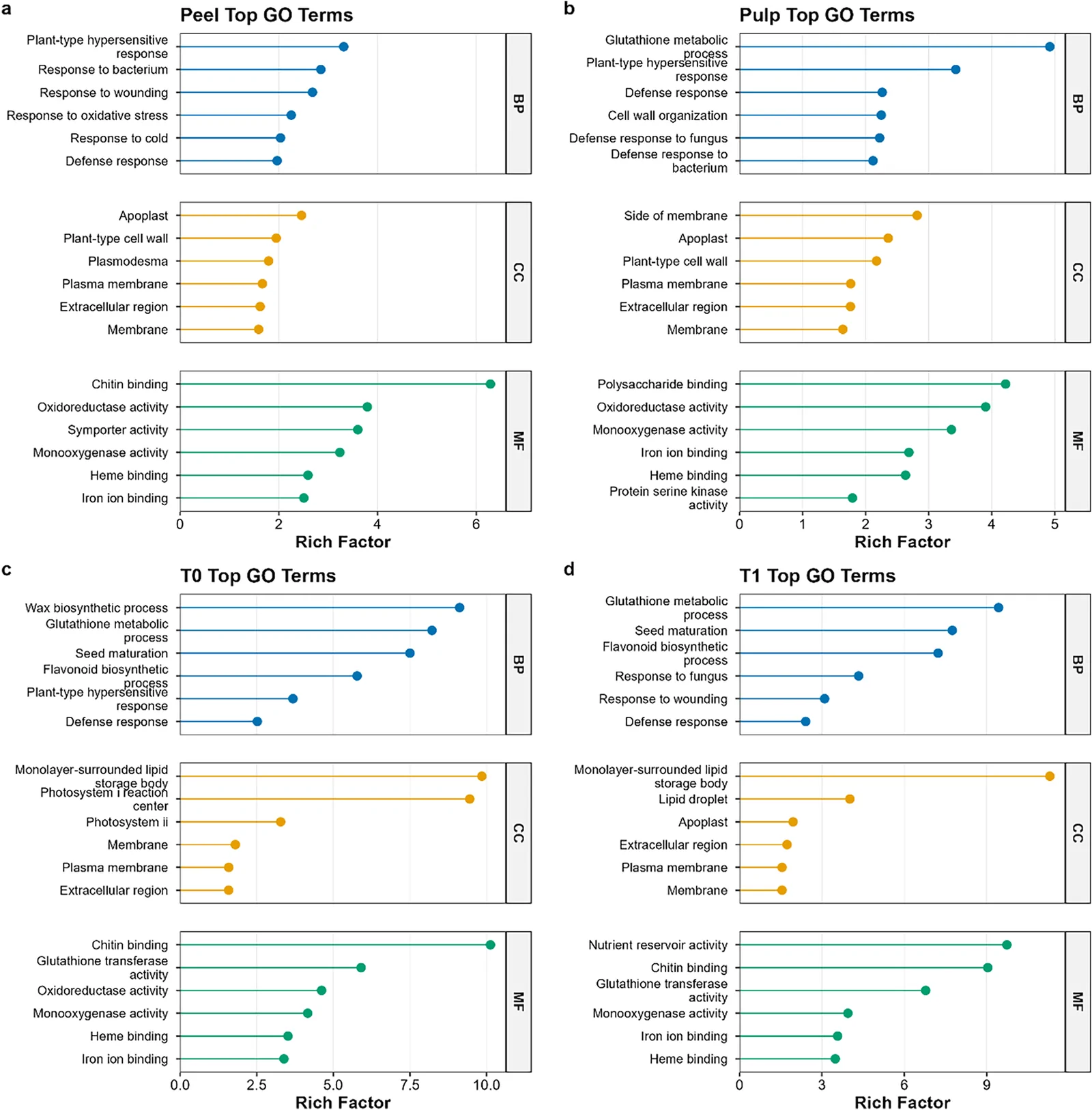
Gene Ontology (GO) enrichment analysis of differentially expressed genes associated with cold storage and tissue-specific transcriptional responses in blueberry fruit. Panels represent GO enrichment of DEGs identified from the following comparisons: (a) peel tissue, cold-stored versus harvest fruit; (b) pulp tissue, cold-stored versus harvest fruit; (c) peel versus pulp at harvest (T0); and (d) peel versus pulp after cold storage (T1). Enrichment analysis was performed using Fisher’s exact test with Benjamini–Hochberg false discovery rate (FDR) correction. Only significantly enriched GO terms (adjusted *p*-value <0.05) are shown. The top eight GO terms within each ontology category—Biological Process (BP), Cellular Component (CC), and Molecular Function (MF)—are displayed. Lollipop plots depict the Rich Factor, calculated as the ratio of the proportion of DEGs annotated to the GO term relative to the proportion of background genes annotated to the same term, with higher values indicating stronger enrichment.

### 3.5 Integration of Transcriptomic and Metabolomic Responses

Integrated multi-omics analysis using DIABLO and MOFA2 identified storage duration and tissue type as the main sources of molecular variation in cold-stored blueberries. DIABLO revealed a strong correlation between transcriptomic and metabolomic latent components (*r* = 0.99; Figure S7), indicating strong covariation between the two omics layers. MOFA2 identified storage duration as the dominant latent factor (Factor 1), explaining most variation across both layers (Figure S8). Both methods also separated samples based on tissue types, with peel demonstrating larger shifts along the storage-associated factors than pulp samples (Figures S6, S7). Gene–metabolite network analysis further revealed highly connected molecular responses during cold storage (Figure S9). Integration across DIABLO, MOFA2, and network analyses identified a consensus set of 65 genes and 58 metabolites (Tables S4–S5).

Hierarchical clustering analysis of 65 consensus genes grouped into six major clusters (Figure 7; Table S4). Cluster 1 comprises genes induced during cold storage in both tissues, which were associated with jasmonate signaling, oxylipin (lipid-derived signaling molecules) biosynthesis, defense responses, and ABC transporters. Clusters 2 and 6 contained genes preferentially expressed in the peel, and were enriched for pathways involved in flavonoid biosynthesis and cuticle lipid metabolism. Clusters 3 and 4 comprised genes expressed at higher levels in the peel at harvest and induced in both tissues during storage, including genes associated with the mevalonate pathway, terpenoid metabolism, polyol transport, defense-related processes, and WRKY transcription factor regulation. In contrast, Cluster 5 contained genes that were consistently downregulated during storage, including cell wall-modifying enzymes, aquaporin PIP1–2, and sterol methyltransferase SMT1. The 58 consensus metabolites belonged primarily to terpenoids, lipids, lignans, coumarins, and flavonoids (Table S5; Figure 8). Clustering analysis grouped these metabolites into five major clusters. Cluster 1 consisted of abscisate and desacetoxymatricarin, both of which decreased during storage in peel and pulp tissues. Cluster 2 contained lipid-derived metabolites, including LysoPC 18:4 and oxylipin-related hydroperoxides such as 9­(S)-HPODE and 13­(S)-HPOT, together with coumarins including isofraxidin, isoscopoletin, and scopoletin. These metabolites increased during storage in both tissues, although their abundance remained higher in the peel. Clusters 3 and 4 were dominated by terpenoids, together with selected lipids and lignans, that accumulated predominantly in the peel and generally increased during storage. Cluster 5 comprised a mixture of terpenoids, lipids, phenylpropanoids, lignans, and coumarins, characterized by the storage-induced accumulation of coniferyl alcohol and LysoPE 16:1.

**Figure 7:**
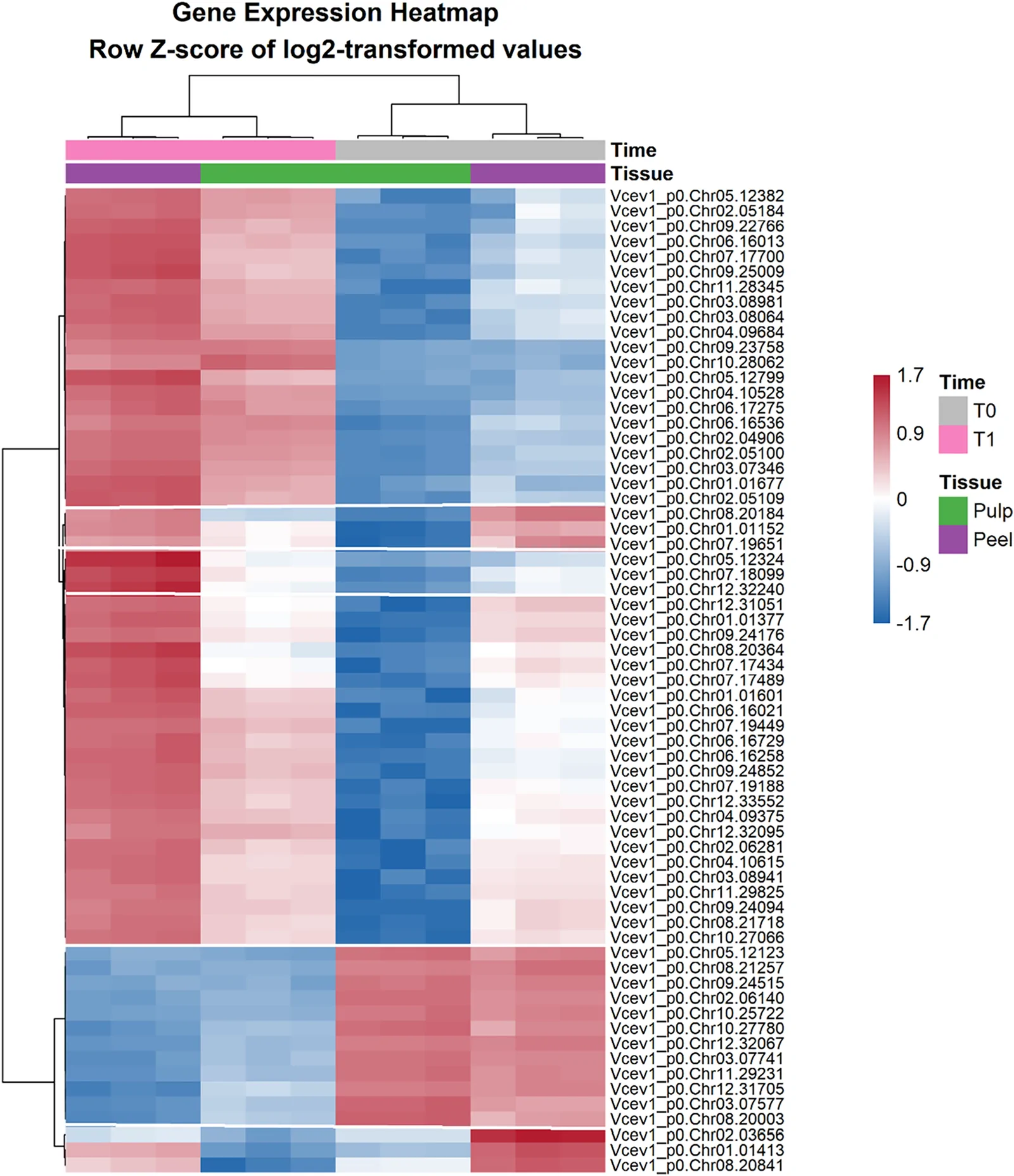
Hierarchical clustering heatmap of consensus genes identified through multi-omics integration. Heatmap visualization of 65 consensus genes identified through integrative transcriptomic–metabolomic analysis. Gene expression values were log_2_-transformed, row-wise Z-score normalized, and hierarchically clustered using Pearson correlation distance and Ward’s D2 linkage method. Samples were clustered using the same approach. Column annotations denote tissue type (peel and pulp) and storage time (T0 and T1). Red and blue indicate relatively high and low expression levels, respectively.

**Figure 8:**
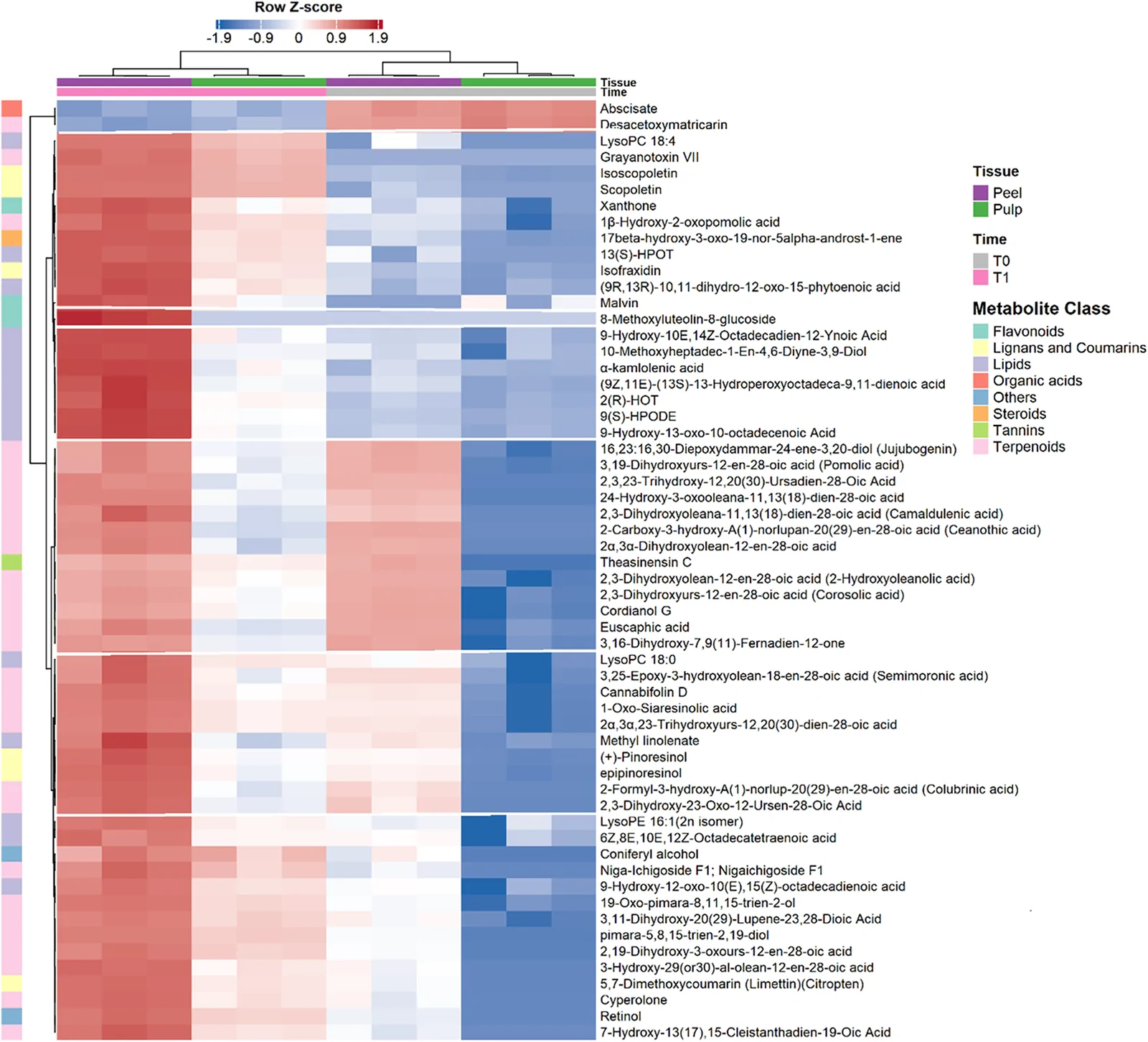
Hierarchical clustering heatmap of consensus metabolite features identified through multi-omics integration. Heatmap of 58 consensus metabolites identified through integrative transcriptomic–metabolomic analysis. Metabolite abundances were row-wise Z-score normalized and hierarchically clustered using Pearson correlation distance and Ward’s D2 linkage method. Column annotations denote tissue type (peel and pulp) and storage time (T0 and T1). Red and blue indicate relatively high and low metabolite abundances, respectively.

Correlation analysis of module eigengenes demonstrated significant associations between gene and metabolite clusters (Figure S10). Gene cluster 1 showed a positive association with metabolite clusters 2 and 5. Gene clusters 3 and 4 were associated with metabolite cluster 4, whereas gene cluster 5 was positively correlated with metabolite cluster 1.

## 4 Discussion

Understanding how distinct fruit tissues coordinate their responses during postharvest storage is essential for revealing the molecular mechanisms underlying quality retention and cold adaptation. By integrating tissue-resolved transcriptomic and metabolomic analyses, this study provides a comprehensive view of the molecular processes underlying blueberry storage responses. This study revealed that peel and pulp exhibit distinct metabolite and gene-expression profiles at harvest, consistent with their specialized physiological functions. These tissue-specific characteristics persist throughout storage, even as both tissues activate broadly conserved responses involving redox regulation, amino acid metabolism, lipid remodeling, and stress signaling. Integrated multi-omics analyses further revealed coordinated gene–metabolite networks that identify candidate pathways associated with storage adaptation. Together, these results support a model in which tissue-specific molecular specialization establishes the physiological framework for postharvest responses, while coordinated storage-responsive pathways contribute to maintaining fruit quality during prolonged storage.

### 4.1 Tissue-Specific Metabolite and Gene-Expression Profiles at Harvest Define Functional Differences between Peel and Pulp

At harvest, blueberry peels and pulp exhibit distinct metabolite and gene-expression profiles consistent with their different physiological functions. The peel is enriched in flavonoids, phenylpropanoids, terpenoid-related metabolites, cuticle-associated lipids, and transcripts involved in cutin, wax, and fatty acid metabolism. This enrichment is consistent with a protective role of the peel in defense and cuticle formation.^36,37^ Similar enrichment of secondary metabolites and cuticle-related pathways in epidermal tissues has been reported in other fleshy fruits, underscoring the conserved role of fruit surface tissues in environmental protection and defense.^21,23,38–40^ In contrast, the pulp accumulates higher levels of soluble sugar-related metabolites and amino acid derivatives, consistent with its roles in carbon storage, energy metabolism, and fruit quality. Such metabolomic specialization complements the defense function of the peel, indicating functional specialization of the tissues.^21,39^

These tissue-specific molecular signatures define the baseline for the storage responses. During storage, the peel and pulp retain their distinct molecular identities while undergoing coordinated transcriptomic and metabolomic changes. Protective secondary metabolism remains prominent in the peel, whereas primary metabolism remains dominant in the pulp, indicating functional partitioning that may support tissue integrity and metabolic homeostasis during storage.^21,39,40^ This clear molecular separation at harvest provides a critical baseline for interpreting tissue-specific responses to prolonged storage, including both shared and tissue-specific mechanisms.

### 4.2 Storage-Induced Conserved Molecular Responses while Maintaining Tissue Identity

Both the peel and pulp undergo substantial and overlapping molecular changes during storage. More than half of storage-responsive genes were shared between the two tissues, and storage accounted for a major source of transcriptomic and metabolomic variation, indicating that storage is a dominant factor shaping molecular responses, while tissue identity is largely maintained. Similar coordinated postharvest responses have been reported across fruit species.^40–42^

Metabolomic enrichment analysis exhibited that amino acid metabolism, glutathione metabolism, oxidoreductase activity, and UDP-glycosyltransferase-associated processes were enriched in both tissues, consistent with coordinated regulation of redox balances, antioxidant response, and metabolic modification during storage.^20,21,39,41^ Despite these shared responses, tissue-specific enrichment was evident. The peel retained enrichment in lipid- and cuticle-related pathways, whereas the pulp showed greater enrichment in cell wall organization and defense-related pathways, indicating distinct structural and metabolic responses between tissues.

Transcriptomic and metabolomic responses were not completely synchronized between the peel and pulp. Although both tissues showed similar numbers of storage-responsive genes, the pulp exhibited more differentially abundant metabolites and a higher proportion of increasing metabolites, whereas multivariate analyses revealed a stronger storage-associated separation of peel samples. This divergence indicates that transcriptomic changes and metabolite accumulation capture complementary aspects of the storage response rather than directly parallel processes.^43^ In the peel, transcriptomic changes were particularly associated with lipid metabolism and defense-related pathways, whereas metabolite changes in the pulp reflected broader metabolic adjustments during storage. Thus, storage responses are better interpreted at the pathway level than by the number of differentially expressed genes or accumulated metabolites alone.

### 4.3 Integrated Multi-Omics Reveals Coordinated Gene-Expression and Metabolite Changes during Storage

Integration of transcriptomic and metabolomic data sets using DIABLO and MOFA2 identified storage duration and tissue type as major sources of molecular variation and revealed coordinated gene–metabolite patterns associated with blueberry storage responses.

DIABLO showed strong concordance between transcriptomic and metabolomic latent components (*r* = 0.99), while MOFA2 identified the storage duration as the dominant source of variation across molecular layers. Together with network analysis, these complementary approaches identified 65 consensus genes and 58 metabolites that captured key molecular features of the blueberry storage response.

The integrated analysis highlighted coordinated changes in lipid-derived signaling, membrane remodeling, defense, and secondary metabolisms. Genes involved in jasmonate signaling, oxylipin biosynthesis, defense responses, and ABC transporters were associated with oxylipin-related metabolites, coumarins, and lysophospholipids, consistent with coordinated stress signaling and membrane adjustment during storage.^44–49^ Coumarin accumulation may contribute to antioxidant capacity, whereas increased lysophospholipids are consistent with membrane lipid remodeling under stress.^50–52^ In the peel, associations involving the mevalonate pathway, terpenoid metabolism, WRKY regulation, flavonoid biosynthesis, and cuticle-related genes further emphasized the continued importance of secondary metabolism and surface defense during storage. Increased coniferyl alcohol also supported the continued phenylpropanoid activity. In the peel, associations involving the mevalonate pathway, terpenoid metabolism, WRKY regulation, flavonoid biosynthesis, and cuticle-related genes further emphasized the continued importance of secondary metabolism and surface defense during storage. Increased coniferyl alcohol also supported continued phenylpropanoid activity.^53–55^ These relationships should be viewed as coordinated molecular responses rather than as direct regulatory interactions.

The integrated analysis also identified changes in structural and cellular maintenance processes. Downregulation of PIP1–2, SMT1, and cell expansion-related genes in Cluster 5, together with associated metabolite changes, suggested alterations in membrane transport, sterol metabolism, and cell expansion during storage. These molecular changes were accompanied by measurable shifts in fruit mechanical properties. Of the 18 texture parameters evaluated, F1MM, FM, SFM, AFM, LDFM, AIF, MIF, and NIP decreased during storage, whereas DFM and BuStr increased. The magnitude of these changes varied substantially among parameters, with fold changes ranging from approximately 1.0- to 6.9-fold. The largest decreases were observed for Young’s modulus (6.92-fold), YM20 (5.52-fold), and NIP (4.09-fold). YM20 has been reported to correlate strongly with sensory perception of fruit firmness, highlighting its relevance to postharvest changes in fruit texture.^26^ The overall decline in texture parameters agrees with previous reports of reduced blueberry firmness and altered mechanical properties during storage,^13,24^ providing phenotypic evidence of structural deterioration.

Because texture was assessed at the whole-fruit level and molecular responses were resolved by tissue, direct gene–texture correlations could not be established. We therefore used previously identified genome-wide association study (GWAS) and quantitative trait loci (QTL) regions as an independent genetic framework for evaluating the biological relevance of the multiomic candidates.^25,56^ Twelve of the 65 consensus genes overlapped or occurred near regions associated with texture-related traits (Table S4). These genes were enriched in functions related to cell wall organization and loosening, polysaccharide and cellulose turnover, lignin biosynthesis, and cellular water balance, providing a plausible molecular basis for changes in tissue mechanical integrity during storage.

Among these candidates, CCoAOMT provided particularly strong convergence across the molecular, genetic, and functional evidence. *CCoAOMT* (Vcev1_p0.Chr09.24094*)* was upregulated in pulp during storage and mapped within a major locus associated with Young’s modulus in blueberry.^26^ Its role in phenylpropanoid and lignin biosynthesis, together with evidence that VcCCoAOMT5 influences lignin accumulation, anthocyanin metabolism, and fruit firmness in highbush blueberry,^57^ further supports its potential involvement in fruit mechanical properties. The cell-wall-related genes identified in this study include endoglucanase 6 (endo-1,4-β-glucanase/Cellulase) and mannan endo-1,4-β-mannosidase (endo-β-mannanase), which are involved in the modification and degradation of cell wall polysaccharides. Endoglucanases cleave 1,4-β-glucan linkages within cellulose and associated matrix glycans and have been associated with cellulose microfibril disassembly and fruit softening during postharvest storage in apple.^58^ Similarly, endo-β-mannanase hydrolyzes β-1,4 linkages in galactomannans and glucomannans and has been associated with hemicellulosic network disassembly and cell wall loosening in postharvest tomato tissues.^59^ The identification of these genes within genomic regions associated with fruit mechanical traits provides additional support for the potential involvement of cell wall remodeling in blueberry texture changes. However, because these functional relationships have been primarily demonstrated in other fruit species, their specific roles in regulating blueberry tissue integrity and softening during cold storage remain to be experimentally determined.

Overall, the strong transcriptome–metabolome coordination observed by DIABLO, together with the MOFA2 storage-associated factor and gene–metabolite networks, indicates that blueberry adaptation to cold storage involves interconnected changes in defense signaling, lipid remodeling, secondary metabolism, redox regulation, and structural maintenance. The concurrent changes in texture and the overlap of multiomic candidates with GWAS and QTL regions further suggest that these molecular responses are linked to the genetic and phenotypic basis of postharvest quality. Although these associations do not establish causality, their convergence identifies biologically meaningful candidates and pathways for functional validation and provides a framework for understanding and ultimately improving blueberry storage quality.

Overall, these findings demonstrate that blueberry postharvest responses reflect the interplay between tissue-specific metabolic specialization and shared storage-responsive molecular programs. The peel retains molecular characteristics associated with protective barrier functions, whereas the pulp maintains features related to primary metabolism and fruit quality, with integrated multi-omics revealing coordinated changes in stress signaling, lipid remodeling, redox regulation, and structural processes during storage. The identified genes and metabolites provide candidates for future validation and potential molecular indicators of storage responses. However, interpretation is constrained by the use of a single cultivar and whole-fruit texture measurements, which precluded direct tissue-specific molecular–texture relationships. Thus, these associations represent testable hypotheses rather than causally established mechanisms or validated biomarkers. Studies using diverse germplasm, additional physiological measurements, and functional validation will help clarify their broader biological and practical relevance. Collectively, these findings support a model in which blueberry storage performance results from coordinated tissue-specific and shared molecular responses rather than a single dominant regulatory pathway.

## Supplementary Material





## Data Availability

Raw RNA-seq data generated in this study have been deposited in the NCBI Sequence Read Archive (SRA) under BioProject accession **PRJNA1464272** (submission ID: **SUB16176978**). Other data generated in this study are available in the manuscript and Supporting Information.

## Notes

Fruit texture parameters; metabolite profiling and multivariate analyses; differential metabolite and gene analyses; multi-omics integration and transcriptome–metabolome correlations; MOFA2 factor scores; integrated gene–metabolite coexpression network; and hierarchical clustering of gene–metabolite cluster correlations (Figures S1–S10) (PDF)
Texture parameters and measurement units; PERMANOVA of blueberry metabolite profiles; metabolite class distributions across peel and pulp tissues; consensus genes prioritized by DIABLO, MOFA2, and network analyses with annotations and GO terms; and consensus metabolites from integrated multi-omics analyses with KEGG IDs and pathway assignments (Tables S1–S5) (XLSX)
