# Supplementary material for: Integrative Multi-Omics Reveals Tissue-Specific Postharvest Changes in Blueberry Fruit
Source: J Agric Food Chem. 2026 Oct 6;74(41):32734–47. doi: 10.1021/acs.jafc.6c13073 (PMC13693449; doi:10.1021/acs.jafc.6c13073)
Supplement: Supplementary file 1 [file jf6c13073_si_001.pdf]

Integrative Multi-Omics Reveals Tissue-Specific Postharvest Changes in Blueberry Fruit

Molla Fentie Mengist[1*], Romit Seth[2], Massimo Iorizzo[2,3]

[1]Agricultural Research Station, Virginia State University, Petersburg, VA 23806 USA
[2]Plants for Human Health Institute, North Carolina State University, Kannapolis, NC 28081, USA
[3]Department of Horticultural Science, North Carolina State University, Raleigh, NC 27607, USA

* Corresponding author: Molla Fentie Mengist mollafentie@gmail.com

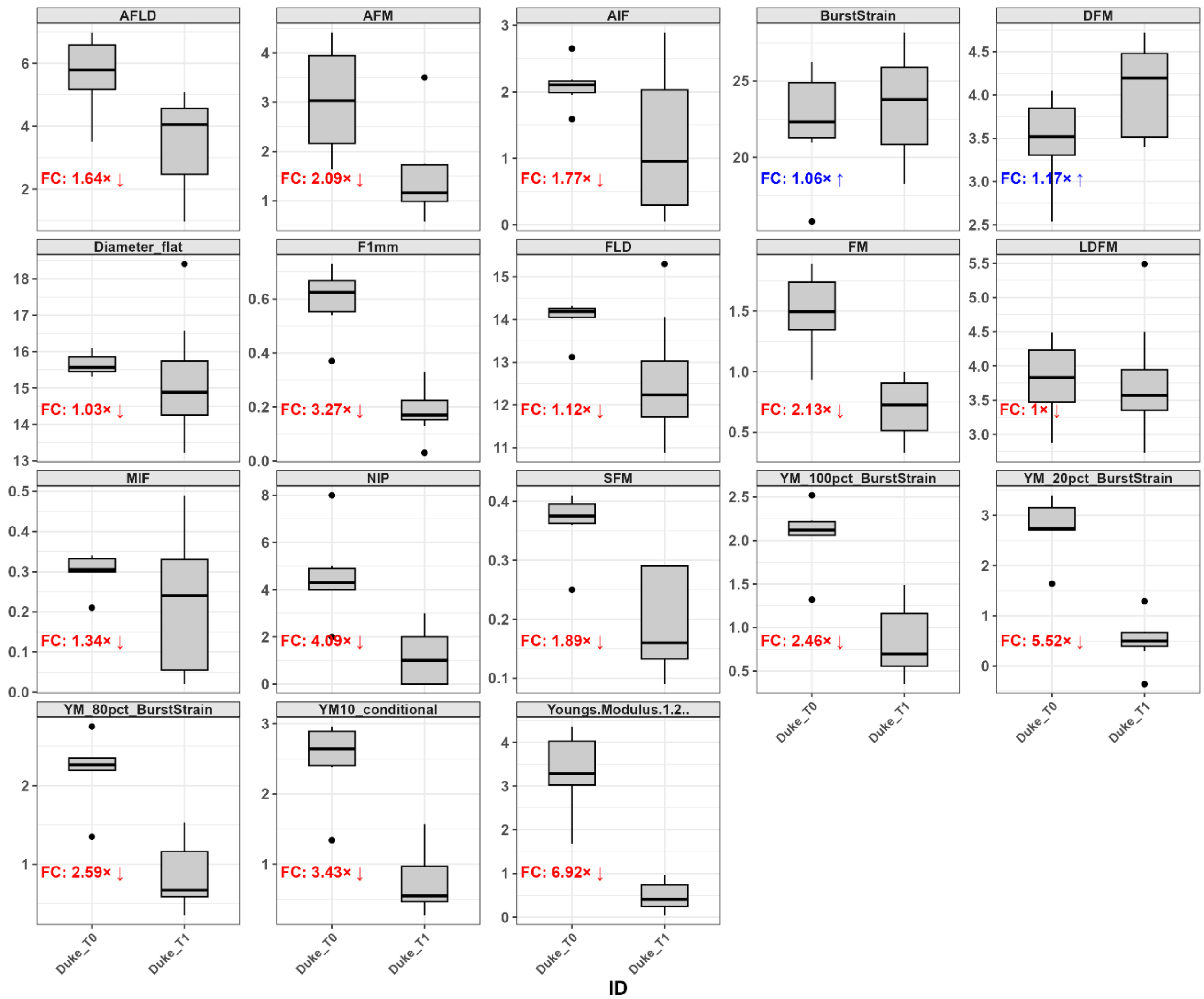

Figure S1. Texture parameters across storage time. Boxplots show the distribution of 18 mechanical texture parameters measured in the Duke cultivar at harvest (T0) and after six weeks of cold storage (T1). Fold change (FC) represents the ratio of the mean value at T1 to that at T0. Red indicates a decrease, whereas blue indicates an increase during cold storage.

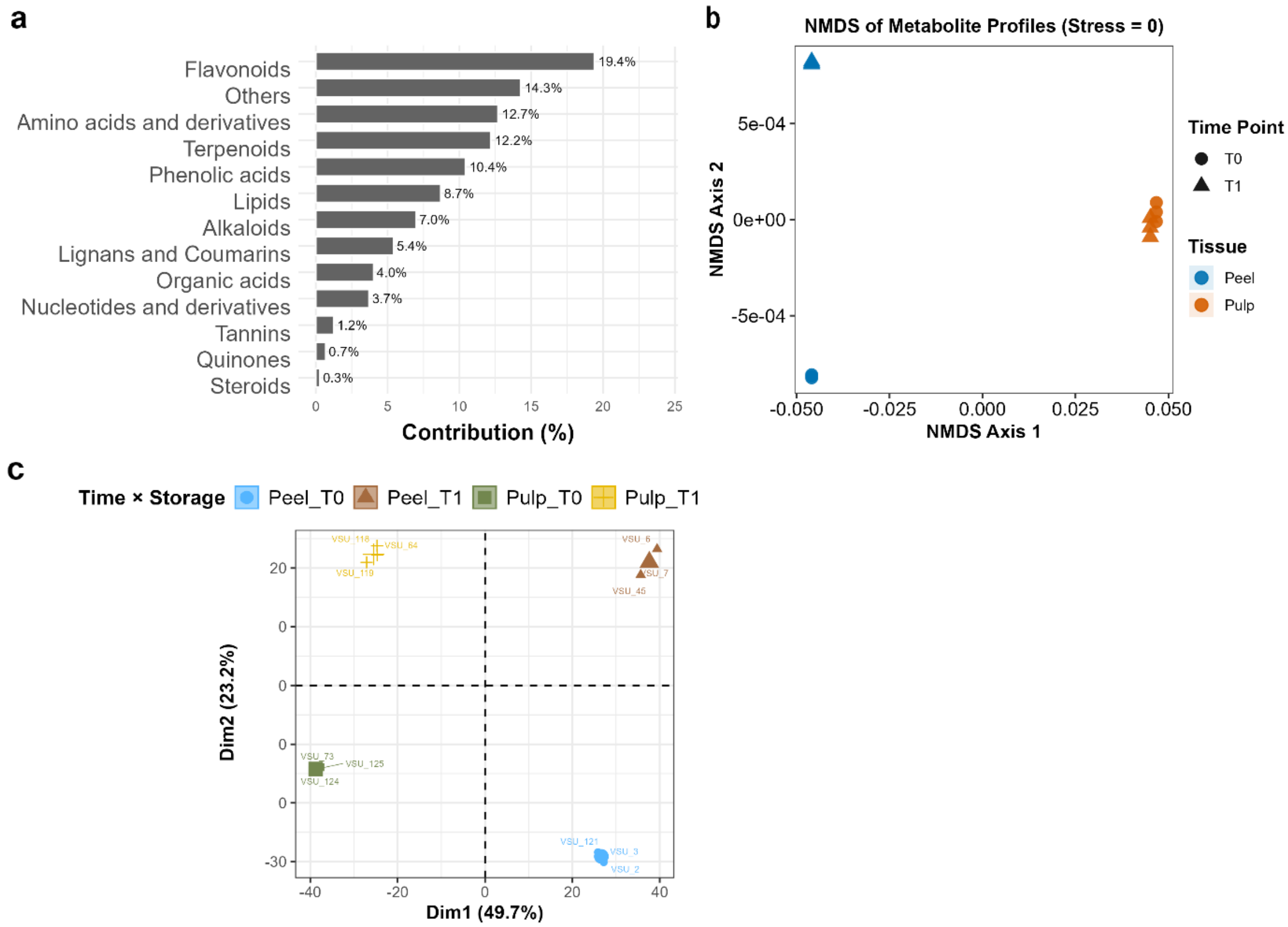

Figure S2. Metabolite profiling and multivariate analysis of blueberry tissues. Metabolite profiles of peel and pulp tissues from the Duke cultivar at harvest (T0) and after six weeks of cold storage at 4 °C (T1). (a) Overview of metabolite identification using widely targeted metabolomic profiling. (b) Non-metric multidimensional scaling (NMDS) of metabolite profiles, with points colored by tissue type and shaped by storage time; convex hulls indicate tissue-specific clustering. (c) Principal component analysis (PCA) of metabolite profiles showing separation by tissue type and storage time.

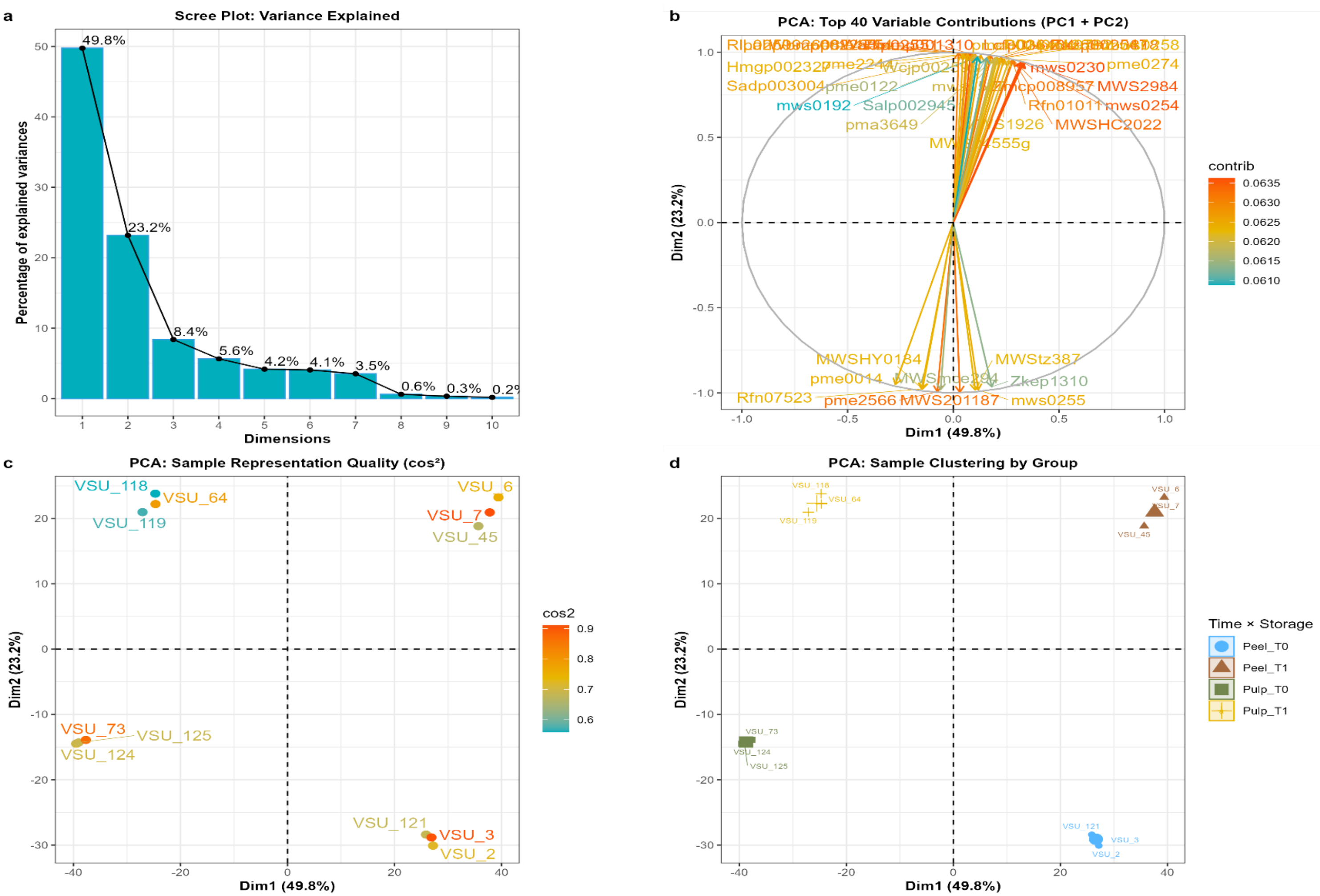

Figure S3. Summary of Principal Component Analysis (PCA) results. (a) Scree plot showing the proportion of total variance explained by each principal component. (b) Contribution of individual variables to the principal components. (c) Score plot displaying the distribution of individual samples in the PCA space. (d) Sample clustering patterns according to tissue type and postharvest storage duration.

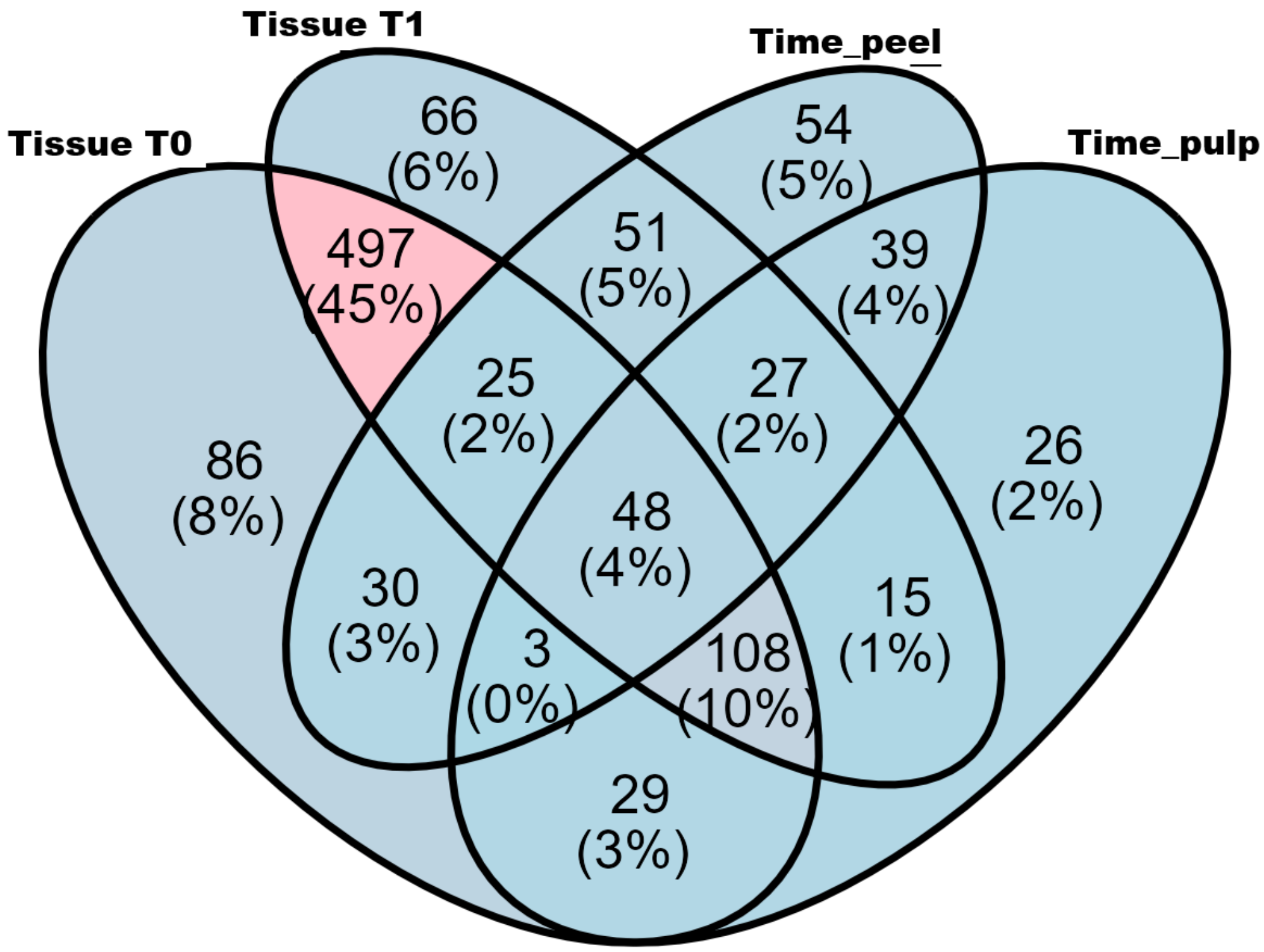

Figure S4. Venn diagram showing the overlap of differentially expressed metabolites (DEMs) across multiple comparisons. DEMs were identified using LIMMA and filtered for adjusted p-value < 0.01 and |log2 fold-change| ≥ 2**.** The diagram highlights shared and unique metabolite regulation patterns between tissues and storage times: Tissue_T0, differences between peel and pulp at harvest; Tissue_T1, differences between peel and pulp after six weeks of cold storage; Time_Peel**,** metabolites altered by storage in peel; Time_Pulp**,** metabolites altered by storage in pulp.

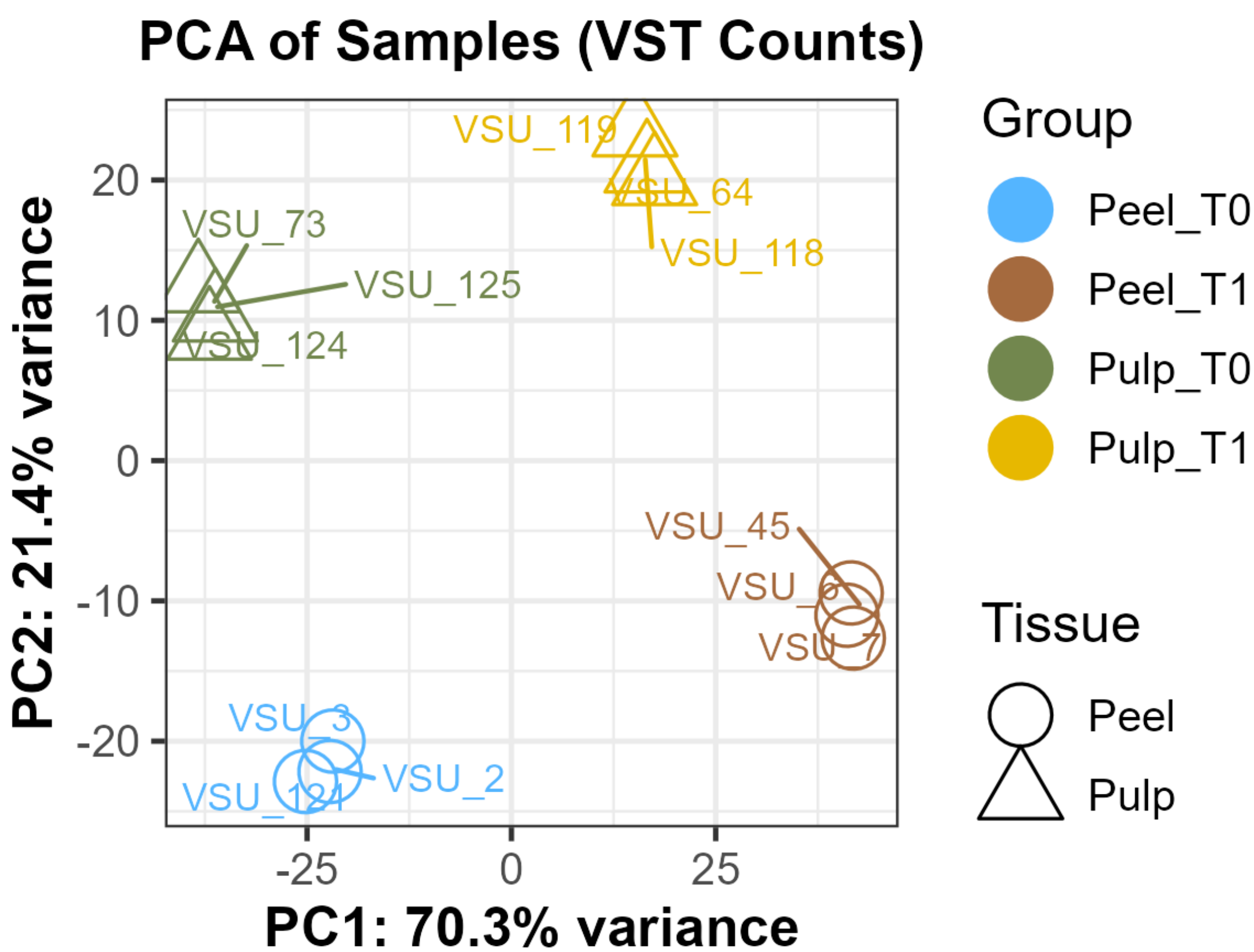

Figure S5. Principal Component Analysis (PCA) of blueberry tissue samples based on variance-stabilized RNA-seq counts. Samples are colored by group (Peel_T0, Peel_T1, Pulp_T0, Pulp_T1) and shaped by tissue type (Peel vs Pulp). The first two principal components are shown, with percent variance explained indicated on the axes. This analysis reveals clear clustering by tissue and storage time, highlighting major sources of transcriptional variation.

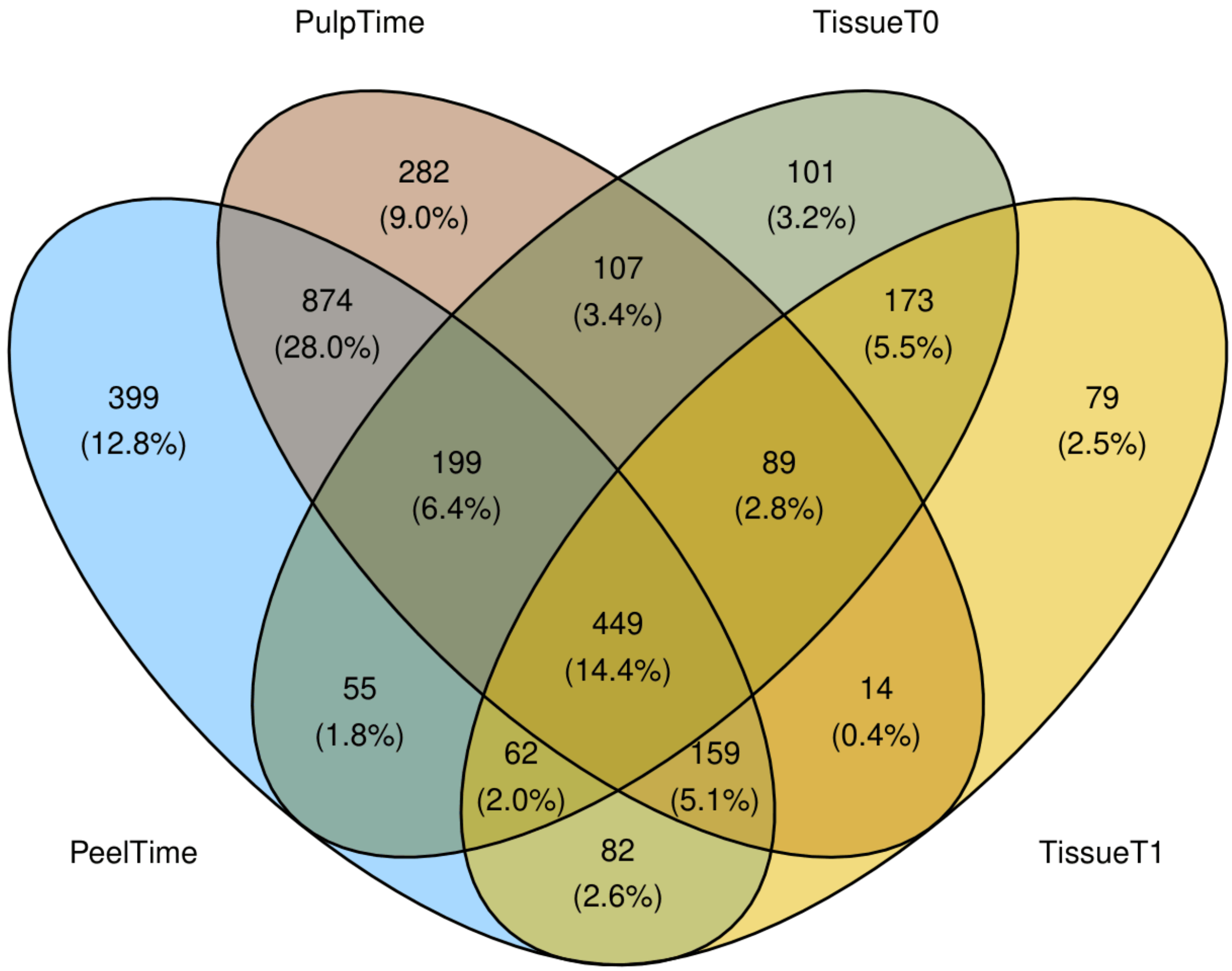

Figure S6. Venn diagram showing the overlap of high-confidence differentially expressed genes (DEGs) across multiple comparisons. DEGs were identified using DESeq2 with apeglm log2 fold-change shrinkage and filtered for adjusted p-value < 0.01**,** |log2 fold-change| ≥ 2, and baseMean ≥ 50. The diagram highlights shared and unique gene regulation patterns between tissues and storage times: TissueT0, DEGs between peel and pulp at harvest; TissueT1, DEGs between peel and pulp after six weeks of cold storage; PeelTime**,** DEGs in peel associated with cold storage; PulpTime, DEGs in pulp associated with cold storage.

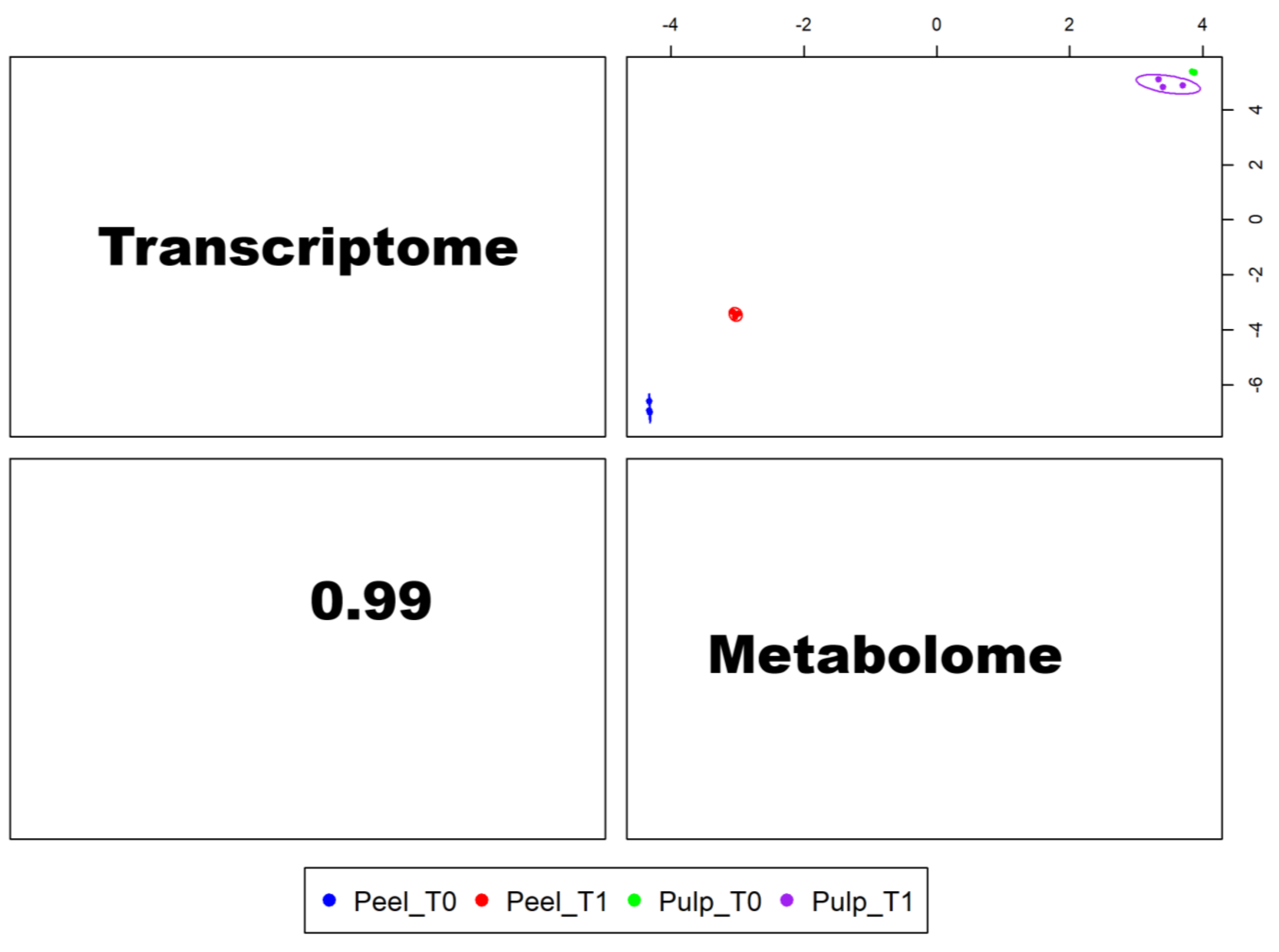

Figure S7. Multi-omics integration and correlation analysis between transcriptome and metabolome profiles. The matrix layout illustrates the multi-block data integration (e.g., via DIABLO or mixOmics framework) comparing transcriptomic and metabolomic datasets across fruit tissue types (Peel vs. Pulp) and time points (T0 vs. T1).

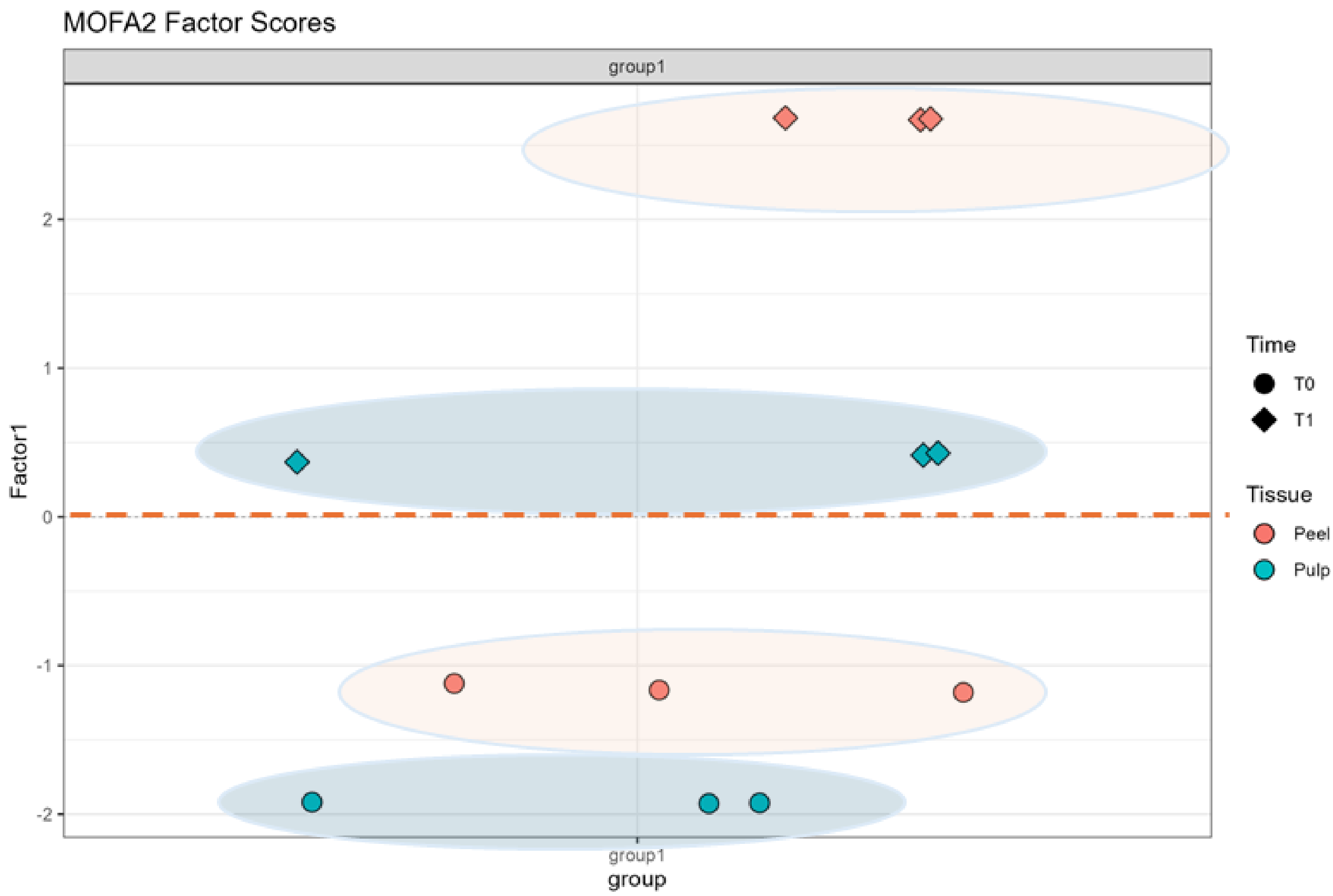

Figure S8. Distribution of MOFA2 factor scores across experimental groups. The scatter plot displays sample factor scores for Factor 1 derived from a Multi-Omics Factor Analysis (MOFA2) model. Y-Axis (Factor 1): Captures variation driving the separation between developmental stages, distinguishing the early time point (**T0**, represented by circles) from the late time point (**T1**, represented by diamonds). Samples clustered below the dashed zero line represent the T0 stage, while samples above it corresponds to the T1 stage.

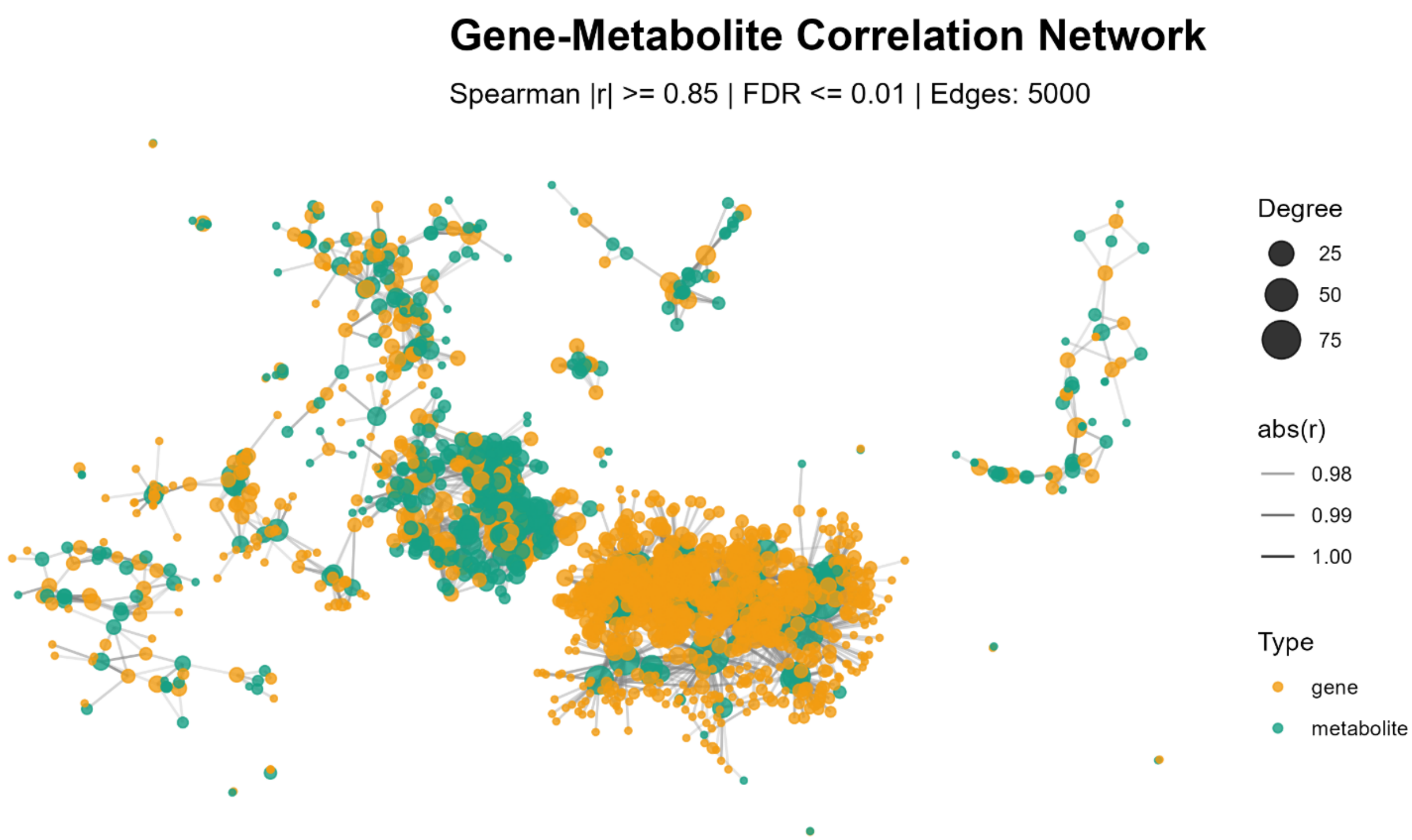

Figure S9. Co-expression and integrated gene–metabolite network. The network illustrates highly correlated associations between transcriptomic and metabolomic features. Network filtering criteria: Edges were retained using a stringent threshold of Spearman's absolute correlation coefficient ($|r| \geq 0.85$) and a false discovery rate (FDR) ≤ 0.01, yielding a network topology comprising 5,000 edges. Node type and color: Nodes are color-coded according to molecular feature class, with genes shown in orange and metabolites in green. Node size (degree): Node size is proportional to connectivity degree (ranging from 25 to >75 connections), highlighting key hub genes and metabolites that contribute substantially to network organization. Edge weight (|r|): Edge thickness and color intensity represent the strength of pairwise correlations, ranging from $|r| = 0.98$ to 1.00. The resulting topology reveals densely connected multi-omics modules, suggesting coordinated regulation and strong functional coupling between transcriptomic and metabolomic components.

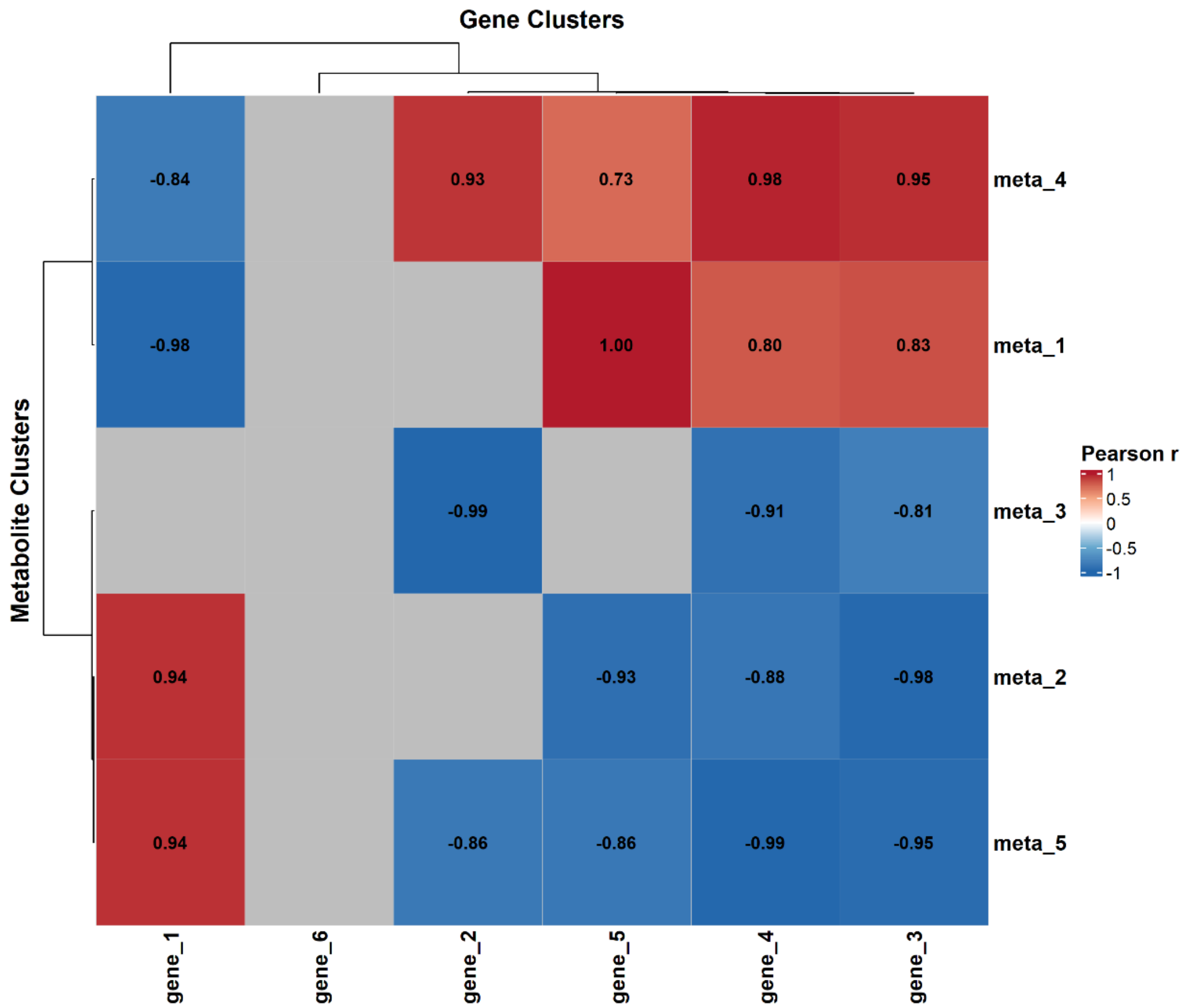

Figure S10. Hierarchical clustering heatmap of correlations between gene and metabolite clusters. The heatmap depicts the correlation structure between gene clusters (columns) and metabolite clusters (rows), revealing coordinated patterns of transcriptomic–metabolomic regulation. Correlation metric and color scale: Cell values and color intensity represent Pearson correlation coefficients (r), ranging from strong negative correlations (dark blue, $r = -1$) to strong positive correlations (dark red, $r = 1$), with grey indicating weak or near-zero associations**.**
